# Assessment of Health Status and Creation of a Registry of Potential Research Participants Aged 1.5 to 50 Years on Bioko Island, Equatorial Guinea

**DOI:** 10.4269/ajtmh.24-0143

**Published:** 2025-04-08

**Authors:** María Silvia Angué López Mikue, Said Abdallah Jongo, Vicente Urbano Nsue Ndong Nchama, Ali Hamad Said, Ali Mtoro, Gertrudis Owono Bidjimi, Marta Alene Owono, Escolastica Raquel Mansogo Maye, Martin Eká Ondo Mangue, Genaro Nsue Nguema Okomo, Beltrán Ekua Ntutumu Pasialo, Dolores Mbang Ondo Mandumbi, Fortunata Lobede Mochomuemue, Juan Carlos Momo Besaha, Raul Chuquiyauri, Kamaka R. Kassim, Ali Mohamed Ali, Ummi Abdul Kibondo, Thabit Athuman, Carlos Cortez Falla, Jeremías Nzamio Mba Eyono, Jordan Michael Smith, Guillermo A. García, José Raso, Elizabeth Nyakarungu, Maxmillian Mpina, Claudia Daubenberger, Mathilde Riloha Rivas, Tobias Schindler, Laurence Lemiale, B. Kim Lee Sim, Marcel Tanner, L. W. Preston Church, Peter F. Billingsley, Stephen L. Hoffman, Thomas L. Richie, Salim Abdulla

**Affiliations:** ^1^ Ministry of Health and Social Welfare, Equatorial Guinea (EGMOHSW), Malabo, Equatorial Guinea;; ^2^ MCD Global Health, Silver Spring, Maryland;; ^3^ Ifakara Health Institute, Bagamoyo, Tanzania;; ^4^ Hospital Regional de Loreto, Loreto, Perú;; ^5^ Swiss Tropical and Public Health Institute (Swiss TPH), Basel, Switzerland;; ^6^ University of Basel, Basel, Switzerland;; ^7^ Sanaria Inc, Rockville, Maryland

## Abstract

A malaria control program established in 2004 on Bioko Island, Equatorial Guinea, achieved significant reductions in malaria prevalence; however, progress subsequently stalled, leading to a proposal to develop and implement a highly effective malaria vaccine to increase impact. After conducting clinical trials of Sanaria® (Rockville, MD) *Plasmodium falciparum* (*P. falciparum*) sporozite vaccines against *P. falciparum* malaria, which demonstrated safety and efficacy, a larger phase 3 trial was planned to support vaccine licensure for specific target populations and eventual island-wide malaria elimination. The Equatorial Guinea Pilot Study for Recruitment, Screening and Participant Registry (EGRESPAR) assessed the health status of the target population and generated a registry of eligible children and adults. Households in areas with malaria prevalence ≥15% were randomly selected, aiming to register 2,100 healthy Equatoguineans divided equally into age groups of 1.5–9, 10–17, and 18–50 years. A total of 6,493 individuals from 1,807 households, who considered themselves or their children to be healthy, completed questionnaires; 2,021 were screened using phase 3 enrollment criteria, 643 were excluded, and 1,378 were entered into the registry. Among those screened, 13.6% had *Plasmodium*, 1.8% had *Loa loa*, 4.8% had hepatitis B, 0.5% had hepatitis C, and 2.1% had HIV. Adults were twice as likely to have clinically relevant medical conditions, such as obesity, hypertension, or diabetes, meeting exclusion criteria compared to children. In conclusion, there was a significant prevalence of infections and chronic medical conditions among Bioko Island residents who considered themselves or their children to be healthy and interested in clinical research participation, particularly in adults. The EGRESPAR successfully generated a registry to support the initiation of a large-scale phase 3 vaccine trial.

## INTRODUCTION

Malaria is a major public health problem in Equatorial Guinea, with *Plasmodium falciparum* (*P. falciparum*) being the most prevalent malaria species in the country and responsible for most malaria-related illness and death worldwide.^1^ The prevalence of *P. falciparum* exceeds 50% in rural areas of the Equatorial Guinea mainland,^2^^,^^3^ which constitutes more than 92% of the land area. Bioko Island, located 23 miles off the coast, is the site of the capital city, Malabo, and was also a high-prevalence area like the mainland^4^^,^^5^ until the implementation of the Bioko Island Malaria Control Project (BIMCP) in 2004 by Medical Care Development International, now MCD Global Health, under, under the supervision of the National Malaria Control Program (NMCP) of the Ministry of Health and Social Welfare of Equatorial Guinea (Ministerio de Sanidad y Bienestar Social de Guinea Ecuatorial [MINSABS]).^6^^–^^8^ The program has focused on indoor residual spraying, the distribution of long-lasting insecticide-impregnated bed nets, larval source management, entomological monitoring, and human case detection and treatment, with these interventions tailored to local transmission characteristics determined by detailed yearly malaria indicator surveys (MISs). After the implementation of the BIMCP, the prevalence of *P. falciparum* parasitemia detected by rapid diagnostic tests in 2- to 14-year-old children living on Bioko Island was reduced from 45% in 2004 to 14% in 2012.^9^^,^^10^ However, progress stalled thereafter, despite continued intensive control efforts, and in 2023, the prevalence was 13.8% (Bioko Island Malaria Elimination Project [BIMEP] MIS, unpublished data).

A consortium consisting of MINSABS, the Equatorial Guinea Ministry of Mines, and several US-based energy companies (Marathon EG Production Limited, Noble Energy, Atlantic Methanol Production Company, and the Equatorial Guinea Liquefied Natural Gas Company) initiated funding for the BIMEP to support the conduct of clinical trials of Sanaria’s (Rockville, MD) aseptic, purified, cryopreserved *P. falciparum* sporozoite vaccines.^11^ Although two *P. falciparum* vaccines based on protein subunits were being developed for use in infants and small children to reduce malaria-related morbidity and mortality,^12^ neither was sufficiently efficacious to address the need for malaria elimination,^13^ and the sporozoite vaccine platform was considered the most promising approach.^11^ The BIMEP consortium included the funders, Sanaria Inc., MCD Global Health, the Ifakara Health Institute (Tanzania), the Swiss Tropical and Public Health Institute (which provided additional funds), and the Malaria Research and Training Center (Mali), with technical support from the WHO. Four studies were conducted on Bioko Island and two in Bagamoyo, Tanzania, to support vaccine development, licensure, and implementation. The four studies on Bioko Island were the Equatorial Guinea Sporozoite Vaccine Trial 1 (EGSPZV1), a randomized, double-blind, placebo-controlled trial (RCT) performed between March and September 2015 involving 33 Equatoguinean men to assess the safety and immunogenicity of the Sanaria® *P. falciparum* sporozoite (PfSPZ) Vaccine (PfSPZ Vaccine, consisting of radiation-attenuated PfSPZ)^14^; the Equatorial Guinea Sporozoite Vaccine Trial 2 (EGSPZV2), an RCT performed between November 2016 and January 2018 involving 119 Equatoguinean adults, children, and infants, to assess the safety and immunogenicity of PfSPZ Vaccine in younger and older age groups, and additionally to compare the efficacy of PfSPZ Vaccine with a second vaccination approach, Sanaria (R) *P. falciparum* sporozoite chemoprophylaxis vaccine (chloroquine) (PfSPZ-CVac (CQ), consisting of non-attenuated PfSPZ combined with administration of the antimalarial chloroquine to attenuate the parasites in vivo) in adults, using controlled human malaria infection (CHMI) to assess efficacy^15^^,^^16^; the Equatorial Guinea Sporozoite Vaccine Trial 3 (EGSPZV3), an RCT performed between August 2018 and April 2019 involving 104 Equatoguinean adults to compare the efficacy against CHMI of four different PfSPZ Vaccine regimens, with the goal of down-selecting the best regimen for ongoing development^17^; and the Equatorial Guinea Malaria Epidemiology Project (EGMALEP), a cleared cohort study performed between January and September 2019 involving 240 adults, children, and infants to measure malaria incidence in preparation for a planned phase 3 trial.^18^ The two trials in Tanzania were the Bagamoyo Sporozoite Vaccine Trial 2 (BSPZV2), an RCT performed between December 2015 and August 2016 involving 93 adults, children, and infants to assess safety and immunogenicity in younger age groups (similar to EGSPZV2) and also to compare the efficacy against CHMI of different immunization regimens of PfSPZ Vaccine in adults,^19^^,^^20^ and Bagamoyo Sporozoite Vaccine Trial 3a (BSPZV3a), an RCT performed between February and July 2018 to assess the safety and efficacy of PfSPZ Vaccine against CHMI in 9 HIV-negative and 12 HIV-positive Tanzanian adults.^21^

These clinical studies added to the findings from trials in several other African countries, demonstrating that PfSPZ Vaccine was safe in African infants, children, and adults.^22^^–^^27^ It provided 78–100% protection against CHMI when administered by optimal routes and schedules,^27^^–^^30^ and it provided 41–86% efficacy against naturally transmitted *P. falciparum* infection in adults in the field.^22^^,^^26^^,^^27^^,^^31^ The studies also demonstrated that the potency of PfSPZ-CVac (CQ) was higher than that of PfSPZ Vaccine,^16^ providing up to 100% protection against both homologous and heterologous strain CHMI at less than one-quarter of the dose.^32^^,^^33^ However, the development of PfSPZ Vaccine remained prioritized over that of PfSPZ-CVac (CQ) for safety reasons.

Based on these results, the BIMEP initiated plans to conduct a randomized, double-blind, placebo-controlled phase 3 trial of PfSPZ Vaccine on Bioko Island. This trial aimed to provide pivotal data on field efficacy to support regulatory submissions to African regulatory agencies, the United States Food and Drug Administration, and the European Medicines Agency, with the goal of licensure and subsequent program integration. The ultimate objective was to initiate mass vaccination programs (MVPs) to eliminate *P. falciparum* from Bioko Island and to immunize frequent travelers to the mainland to prevent reimportation.

The anticipated number of participants in the trial (2,100) was several-fold higher than the numbers in trials previously conducted on Bioko Island, raising questions about the feasibility of recruiting and retaining such a large study population. To address these questions, the present study was designed, called the Equatorial Guinea Pilot Study for Recruitment, Screening and Participant Registry (EGRESPAR). The objectives were to optimize procedures for expanding recruitment, screening, and enrollment; to assess the health status of potential research participants; and to generate a participant registry of eligible individuals for the phase 3 trial. It was intended that the EGRESPAR would sensitize potential research participants and their communities to local PfSPZ vaccine development efforts and promote the potential benefits of an island-wide vaccination program, thereby improving rates of recruitment, screening, and enrollment, as well as study efficiency, participant compliance, and the resulting quality of data.

This study recruited participants from selected areas of Bioko Island, specifically in and around the capital city, Malabo. The enrollment criteria mirrored those anticipated for the phase 3 trial (Supplemental Table 1). Potential research participants were identified using the BIMEP’s mapping system to locate households^34^ and conduct home visits. They were then consented and screened at recruitment venues (RVs; tier 1 screening) and at the clinical research center (CRC; tier 2 screening). This paper reports on the findings from the 2,021 participants screened before changes in development priorities and the severe acute respiratory syndrome coronavirus 2 (SARS-CoV-2) pandemic interrupted the study and halted preparations for the phase 3 trial.

## MATERIALS AND METHODS

### Study area and population.

**Figure 1.**
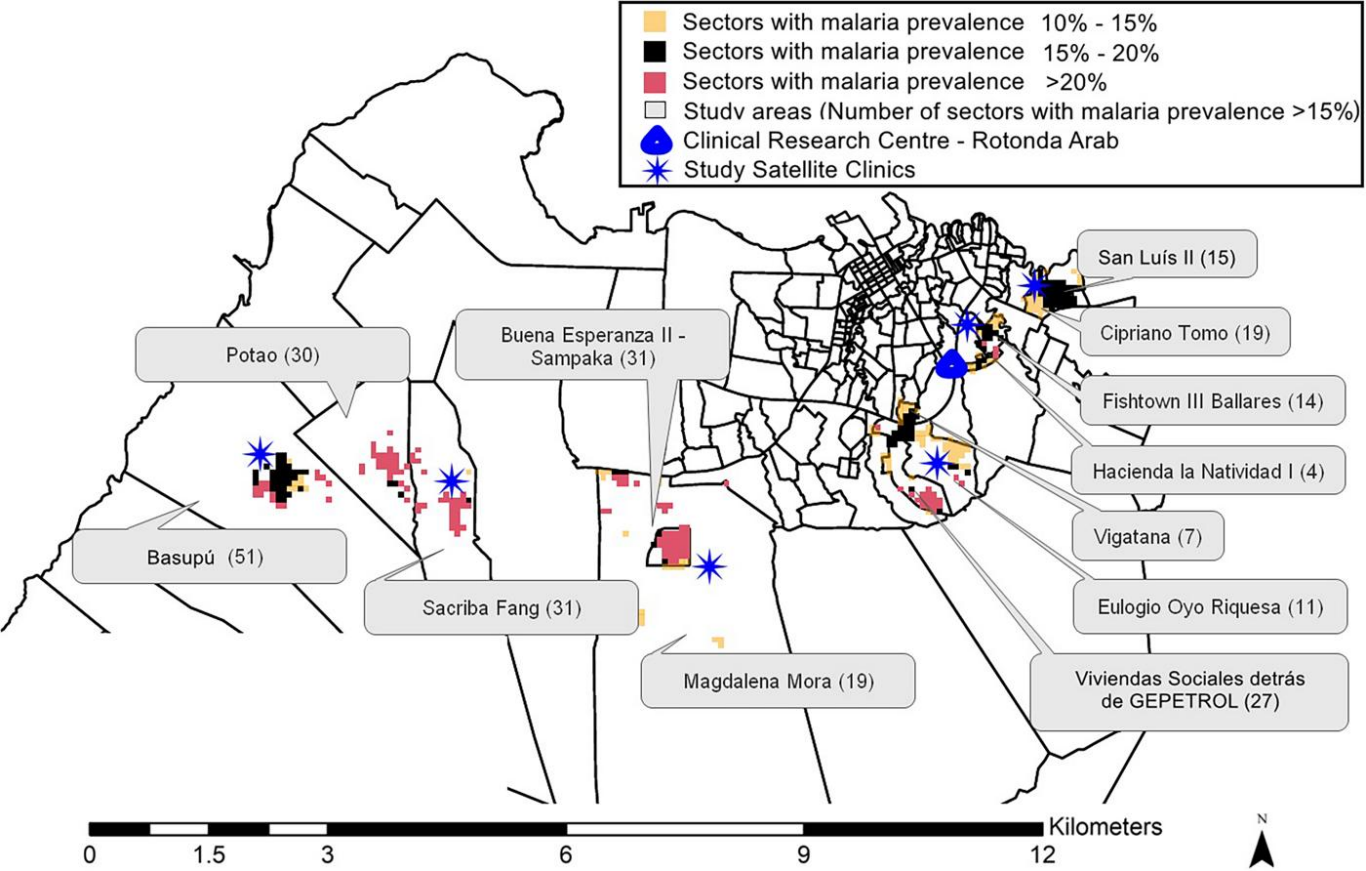
Map of the Equatorial Guinea Pilot Study for Recruitment, Screening and Participant Registry study area showing the locations and (number) of sectors (small squares) containing households that were visited, color-coded by malaria prevalence in 6- to 10-year-olds (see key). All sectors were in 12 administrative areas of Bioko Island (black borders).

The study enrolled healthy Equatoguinean male and female children, adolescents, and adults who were 18 months to 50 years of age at the time of screening. The study population came from localities within 12 administrative areas with relatively high malaria prevalence and a low likelihood that the malaria present in the community was travel-associated^35^ (Figure 1). These communities included Fishtown III Ballares, Hacienda la Natividad I, Cipriano Tómo, San Luis II, Vigatana, Eulogio Oyo Riquesa, Vivienda Sociales detrás de GePetrol, Buena Esperanza II, Magdalena Mora, Sacriba Fang, Potao, and Basupú. The population is composed of several ethnic groups, including Fang, Bubi, Annobones, Ndowe, Bisio, and Fernandino. Permission was obtained from local government officials and community representatives to use the BIMEP/NMCP household database to randomly identify individuals to be invited to participate in the study.^34^ Naturally acquired immunity, which manifests as reduced susceptibility to clinical disease, increases progressively with age in the study population and could potentially affect vaccine trial outcomes, including safety, tolerability, immunogenicity, and efficacy. Therefore, these individuals were stratified into three balanced age groups (1.5–9, 10–17, and 18–50 years) to align with the plan for similar age stratification in the phase 3 trial.

### Study sites.

The EGRESPAR study was headquartered at the BIMEP CRC located in the Rotonda Arab area of Malabo, Bioko Island, but was conducted primarily through household visits and five satellite clinics strategically located in San Luis II, Eulogio Oyo Riquesa, Buena Esperanza II, Potao, and Basupú (Figure 1). Serious illnesses in these areas are generally treated at the government-owned Malabo Regional Hospital. There are also several private hospitals in close proximity to these communities. All five clinical sites were modified to serve as RVs with sufficient office space, a registration desk, a waiting area, clinical examination rooms, a nursing area, space for supplies and storage, and internet access. The CRC site was upgraded to include a pharmacy and document archive. The clinical hematology and chemistry laboratories were located at the newly constructed Baney Research Center (Centro de Investigaciones de Baney), which was launched in February 2019.

### Study design.

The EGRESPAR was a cross-sectional, noninterventional study conducted from September 2019 to March 2020. This single-center study expanded the recruitment and screening approach previously approved by community leaders and used to enroll participants in previous studies.^14^^–^^18^

### Community engagement and sensitization.

Before initiating recruitment, the clinical team met with MINSABS and local community leadership to explain the study and launched information campaigns through radio, television, posters, and flyers. Permission to recruit was obtained from local officials. Subsequently, community meetings were held to sensitize the population to the study. Recruitment was conducted through door-to-door household visits and limited, institutional review board (IRB)-approved preconsent screening (Supplemental Form 1) during these visits to avoid scheduling individuals for consenting at RV visits who were clearly ineligible due to age or general health status. The number of individuals per age category per household that could be entered into the EGRESPAR registry was restricted to two to reduce the potential influence of household-related factors on trial endpoints in a future vaccine trial, including reduced transmission rates should a high proportion of household members receive the test vaccine.

### Selection of study population and rationale.

Study areas were selected in locations on Bioko Island where malaria prevalence was ≥15%, and the parasites appeared to be locally acquired (as opposed to imported by travelers from the mainland), as determined by yearly MISs conducted by BIMEP, which included travel history,^35^ and by the results of a previous incidence study.^18^ The total local population residing within the study areas was 16,557 persons in 3,955 households. The 12 sectors with a malaria prevalence ≥15% included 11,890 persons in 2,786 households (2018 MIS). The Microsoft Visual FoxPro Version 9.0 package (Microsoft Corp., Redmond, WA) was used to randomly select and order households, and then to select and order individuals within each age group in each household. Household selection was constrained to ensure a balanced geographic distribution within each community.

### Participant eligibility and screening.

The goal of the initial home visit was to invite potentially eligible individuals to RVs for informed consent and first-tier screening. Suitability for screening was determined during the home visit using the IRB-approved preconsent screening criteria (Supplemental Form 1 and Supplemental Table 2), which, if met, resulted in an invitation to the local RV in the following days. RV visits began with presentations about the study, followed by informed consent. Adults were asked to provide written consent for themselves or their children; 9- to 17-year-olds were asked to provide written assent, and 6- to 8-year-olds were asked to provide verbal assent. The clinical team then collected sociodemographic and medical history data, measured vital signs (respiratory and pulse rate, systolic and diastolic blood pressure, and axillary temperature), and assessed anthropometric indices, including weight, height, and body mass index (BMI). For children aged 11–17, Z-score BMI for age was calculated, and for children under 11 years of age, Z-score BMI for weight was determined (Supplemental Figures 1 and 2) to evaluate nutritional status. The individual being screened (or the parent or guardian) was notified at the end of the screening visit or later by telephone if he or she (or the dependent child) was excluded from further procedures (Supplemental Tables 3 and 4) or invited to the CRC for second-tier screening (Supplemental Table 4). At the CRC, electrocardiograms (ECGs) and general physical examinations were performed (the latter without pelvic or rectal examinations unless indicated by medical history or other findings). Blood samples were collected for thick blood smear (TBS) to assess malaria parasitemia (also used to screen for *Loa loa* [*L. loa*] or *Mansonella perstans* [*M. perstans*] microfilaremia), hepatitis B (Hexagon HBsAg rapid test for hepatitis B surface antigen, Human Diagnostics Worldwide, Wiesbaden, Germany), hepatitis C (Hexagon HCV rapid test for hepatitis C antibodies, Human Diagnostics Worldwide), and HIV (Determine HIV1-2 Combo Test, Alere, Waltham, MA; Uni-Gold HIV-1/2 Test, Trinity Biotech, Bray, Ireland; and SD Bioline HIV 1/2 Test, Standard Diagnostics Inc., Yongin-si, South Korea, for antibodies to HIV-1 and -2). A complete blood count with differential (neutrophils, lymphocytes, and eosinophils; HORIBA ABX Pentra 60 C+ hematology analyzer, HORIBA, Irvine, CA), along with biochemistry tests including alanine aminotransferase, creatinine, and random blood glucose (Roche Cobas Integra 400 plus chemistry analyzer, Roche, Basel, Switzerland; Supplemental Tables 5 and 6). Positive rapid tests for hepatitis B, hepatitis C, and HIV were confirmed using the Roche Cobas E411 ElectroChemiLuminescence automated analyzer. A urine sample was collected for human chorionic gonadotropin pregnancy testing in females aged 9–45 years. Pre-test (and later post-test) counseling was provided for hepatitis and HIV assessments.

Individuals with a history or clinical manifestations of serious or chronic disease (Supplemental Tables 3, and 4), including hypertension, cardiac disease, diabetes, renal disease, hepatitis B or C, or HIV infection, were excluded, and appropriate healthcare referrals were made for further care. Also excluded were those perceived to have an increased risk of nonadherence to study procedures, including those intending to move from the study area during the anticipated phase 3 study period. Participants with minor illnesses or those found to be positive for malaria, *L. loa*, or *M. perstans* were treated by the study team clinician according to local treatment guidelines and could be included.^36^ Those who met all eligibility criteria were asked if they were willing to have their names and contact information entered into the participant registry.

### Compliance with study visits and procedures.

Longitudinal compliance with study visits and procedures was encouraged through multiple contact methods (phone calls to the participants, home visits by community mobilizers, and phone calls to close contacts as needed). Transportation to the RV or CRC was provided if necessary, along with compensation for attending each visit. A participant received 3,000 Central African Francs (just over $5) in cash for visits to the RVs and the CRC. In addition, light snacks and drinks were provided at the RV and CRC. Payment was made to a parent or guardian in the case of minors, with the stipulation that the payment should be used to support the child’s wellbeing or advancement. In addition, participants were reimbursed for the cost of transportation to the RV or CRC for scheduled visits (but not unscheduled visits) if the participant was unable to use the transport provided by the study. Study doctors were available 24 hours per day, 7 days per week to assist study participants who had signed consent forms and had not yet reached the study’s end, which was defined as the point at which the individual was excluded or, if enrolled, their name was or was not entered into the participant registry.

### Data management.

At the household level, information was collected using the ODK software system (ODK, San Diego, CA, https://getodk.org) using tablets. At the RV and CRC, information was collected on paper-based case report forms, which were then entered into a customized electronic database, Castor EDC^R^ (Castor, Amsterdam, the Netherlands). Data were entered and verified by independent teams.

### Sample size.

This was a descriptive study with no hypothesis testing; hence, the number of participants included in the registry was driven by the anticipated sample size for the phase 3 trial, which required 2,100 research participants. The study originally targeted ∼3,000 individuals, equally divided into three age categories, but this goal was not met because of study interruption (see below).

## STATISTICAL ANALYSES

A descriptive statistical assessment was performed. The number of persons screened, the number of screen failures, and the reasons for screen failure and enrollment were tabulated overall and by age group and sex. Demographic, laboratory, and clinical characteristics were summarized in tables and figures. Frequency tables were used to summarize the distributions of categorical data, whereas continuous data were summarized using the mean, SD, and range. STATA (version 15; StataCorp, College Station, TX) and R Statistical Software (version 3.4.3; R Foundation, Vienna, Austria, https://www.r-project.org/) were used for summary statistics and graphical analysis, respectively.

## RESULTS

### Door-to-door canvassing of households.

**Figure 2.**
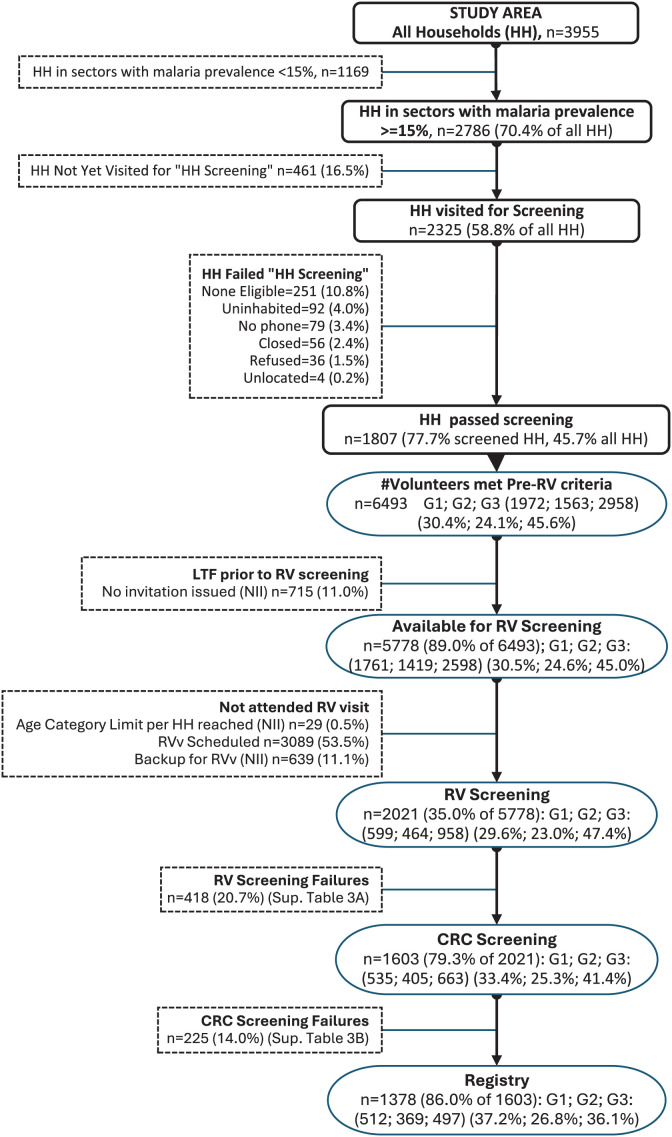
Consort diagram for the household and participant disposition. G1, G2, G3 = Groups 1, 2 and 3 (1.5–9 years, 10–17 years, and 18–50 years of age). LTF = lost to follow-up.

After the randomized, hierarchical sampling frame was used to select households and individuals from the BIMEP/NMCP household database (see Methods), door-to-door canvassing of the preselected households and household members was conducted from September 9, 2019 to February 18, 2020. A total of 6,493 individuals living in 1,807 (77.7%) of the 2,325 preselected households visited during this period expressed an interest in participating (or in their dependents participating) and responded to the IRB-approved preconsent screening questionnaire. In 36 (1.5%) households, there was no interest in participating. An additional 251 (10.8%) households had no eligible volunteer, 92 (4.0%) were uninhabited, 79 (3.4%) had no phone to maintain contact with the study team, 56 (2.4%) were closed, and 4 (0.2%) could not be located (Figure 2).

### Disposition of screened participants at the RV and CRC.

The questionnaires from 6,493 interested individuals were reviewed. A total of 5,110 (78.7%) were invited to the RV for screening, and of these, 2,021 (39.5%) attended the RV and 3,089 (60.5%) remained on the waiting list to attend the RV when the study ended. An additional 715 (11.0%) were invited but were lost to follow-up before the RV visit, 29 (10.3%) were excluded because they would have exceeded their age category limit per household, and 639 were kept in reserve as backups (Figure 2). At the RV visit, all 2,021 attendees, which included 599, 464, and 958 individuals in the 1.5–9 years, 10–17 years, and 18–50 years age categories, respectively, signed consent or provided assent to proceed with screening (Table 1). A total of 418 (20.7%) of the 2,021 failed the screening criteria applicable at the first-tier RV level, accounting for 10.7%, 12.7%, and 30.8% of the total screened in the age categories of 1.5–9 years, 10–17 years, and 18–50 years, respectively (Table 1; Supplemental Table 3). Major reasons for failure included unwillingness to meet contraception requirements (*n* = 110, with 94 in the 18–50 year age category and 16 in the 10–17 year age category), anthropometric parameters outside the specified range (*n* = 107, with 93 cases [87%] in adults), and medical history, symptoms, signs, or laboratory values suggestive of a systemic disorder or chronic illness (*n* = 104, with 78 cases [75%] in adults). An additional 225 (11.1%) failed the second-tier screening criteria at the CRC, accounting for an additional 3.8%, 7.8%, and 17.3% of the total screened in the three age categories (Table 1; Supplemental Table 4). The main reasons for exclusion overall were medical history, symptoms, signs, or laboratory values suggestive of a systemic disorder or chronic illness (*n* = 180, with 138 cases [77%] in adults), positivity for HIV or hepatitis (*n* = 118, with 93 cases [79%] in adults), and a history of arrhythmias, prolonged QT interval, or other cardiac disease (*n* = 30, with 26 cases [87%] in adults). Overall, nonclinical reasons for exclusion, such as not residing in the selected community, not willing or unable to attend the required appointments at the CRC for the next 2 years, and not agreeing to be contacted by telephone or home visit, were equally common in all three age groups (Supplemental Table 3). Clinical reasons, such as anthropometric parameters outside the reference interval, medical history, symptoms, signs, or laboratory values suggestive of a systemic disorder or chronic illness, and not being sufficiently healthy according to clinical judgment based on medical history, examination, and investigations conducted in this study, including positive HIV, hepatitis B virus, or hepatitis C virus test results, were most common in adults (Supplemental Table 4). A total of 1,378 (68.2%) met all eligibility criteria and agreed to be entered into the participant registry, including 512, 369, and 497 individuals in the 1.5–9 years, 10–17 years, and 18–50 years age categories, respectively. These figures represent 85.5%, 79.5%, and 51.9% of the total screened in these categories (Figure 2; Table 1).

**Table 1:** Disposition of the 2,021 participants who provided consent for screening

Description	Total	Age Groups (years)		
		1.5–9	10–17	18–50
Provided consent	2,021	599	464	958
Eligible at recruitment venue	1,603 (79.3)	535 (89.3)	405 (87.3)	663 (69.2)
Eligible at clinical research center	1,395 (87.0)	518 (96.8)	373 (92.1)	504 (76.0)
Entered in EGRESPAR registry	1,378 (98.8)	512 (98.8)	369 (98.9)	497 (98.6)

EGRESPAR = Equatorial Guinea Pilot Study for Recruitment, Screening and Participant Registry. Values are *n* (%); denominators for calculating % in each row are the *n* numbers in the row above.

### Health data from RV screening results.

**Figure 3.**
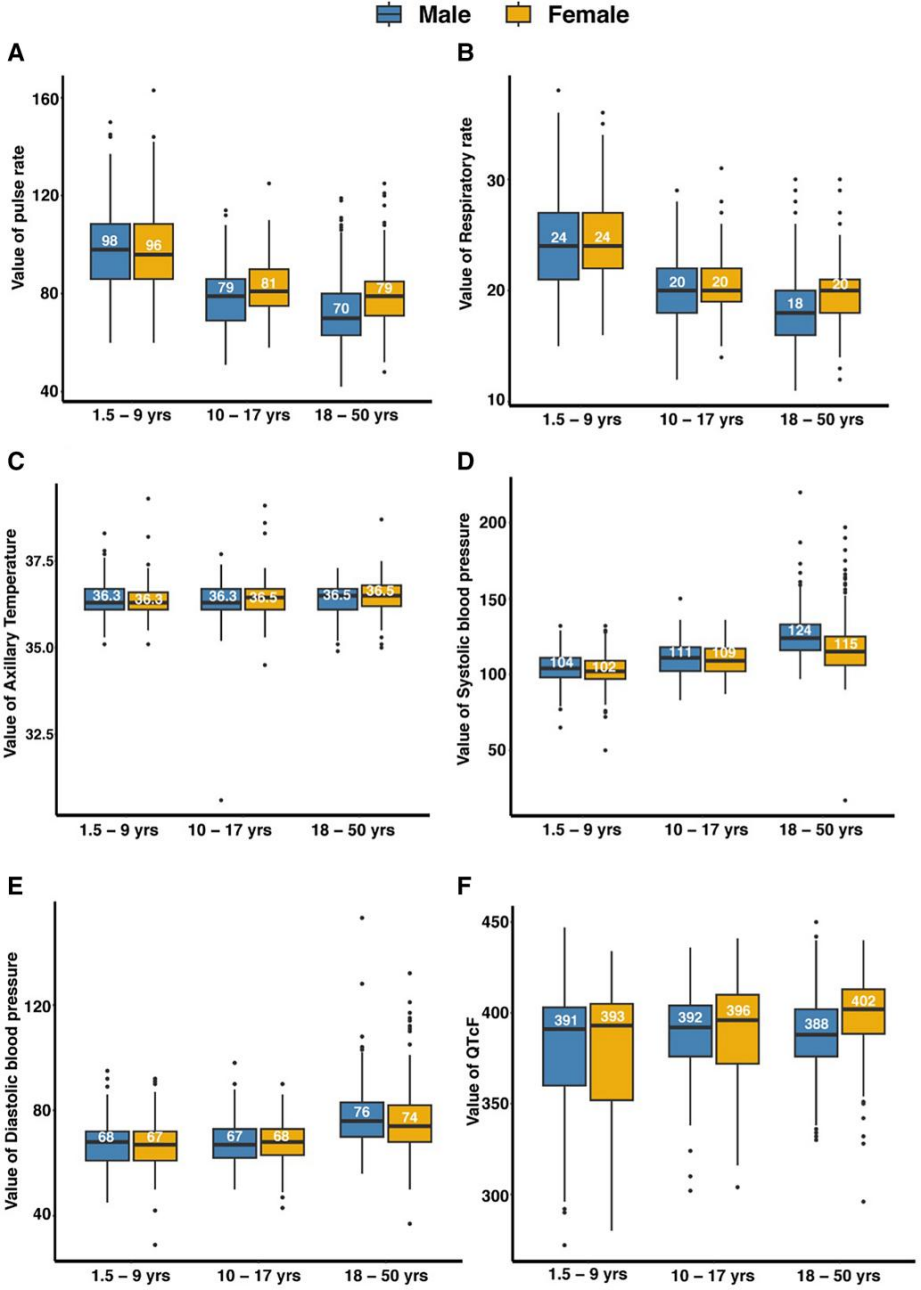
Distributions of clinical parameters of volunteers participated in the Equatorial Guinea Pilot Study for Recruitment, Screening and Participant Registry (EGRESPAR), stratified by age and sex. Panels show the distribution of (**A**) pulse rate (beats/minute), (**B**) respiratory rate (breaths/minute), (**C**) axillary temperature (°C), (**D**) systolic blood pressure (mm of Hg), (**E**) diastolic blood pressure (mm of Hg), and (**F**) QT intervals corrected for heart rate using Fridericia’s formula (QTcF), in milliseconds. The boxes show the median and the upper and lower quartiles, and the whiskers define maximum and minimum values, excluding outliers. Hg = mercury.

Any abnormalities in vital signs (heart rate, respiratory rate, blood pressure, temperature) and anthropometric measurements (body mass index and body mass index/weight for age) from the 2,021 participants undergoing tier 1 screening were assessed for clinical significance by study clinicians and reviewed against age-relevant reference intervals (Supplemental Figures 1 and 2) and protocol eligibility criteria (Supplemental Table 1). The distributions of clinical parameters (pulse rate, respiratory rate, axillary temperature, systolic and diastolic blood pressure, QTcF) are presented in Figure 3. The numbers of individuals with clinical parameters sufficiently outside the reference interval to be deemed clinically relevant for exclusion were as follows: elevated pulse rate 16 (0.8%), elevated respiratory rate 11 (0.5%), elevated axillary temperature 8 (0.4%), elevated systolic blood pressure 3 (0.2%), elevated diastolic blood pressure 5 (0.3%), elevated systolic and diastolic pressures 30 (1.5%), and abnormal ECG 22 (1.4%). Findings for each age category are provided in Table 2. The number of participants outside the reference interval and deemed clinically relevant for exclusion for each anthropometric parameter is provided in Table 3. For BMI among the adult group, 93 (9.7%) had BMI values outside the reference interval. Four (0.4%) had values below the lower limit (18 kg/m^2^), and 89 (9.3%) had values above the upper limit (30 kg/m^2^). Among the children, 14 (1.3%) had a Z-score (BMI for age [children aged 11 years to 17 years] or weight for age [children below 11 years]) outside the reference interval. Two (0.2%) had values less than two standard deviations below the mean, and 12 (1.1%) had values more than two standard deviations above the mean (Supplemental Figures 1 and 2).

**Table 2:** Volunteer numbers (%) with clinical parameters outside reference interval at recruitment venue screening and with ECG parameters outside reference interval (with or without clinically significant abnormalities) at clinical research center screening

Parameter	Group	Total = 2,021 (936 male, 1,085 female)		Age Group					
				1.5–9 Years, *n* = 598 (284 male, 314 female)		10–17 Years, *n* = 465 (214 male, 251 female)		18–50 Years, *n* = 958 (438 male, 520 female)	
		NCS, *n* (%)	CS, *n* (%)	NCS, *n* (%)	CS, *n* (%)	NCS, *n* (%)	CS, *n* (%)	NCS, *n* (%)	CS, *n* (%)
Pulse rate	All	335 (16.6)	16 (0.8)	191 (31.9)	9 (1.5)	51 (11.0)	1 (0.2)	93 (9.7)	6 (0.6)
	Male	160 (17.1)	10 (1.1)	87 (30.6)	7 (2.5)	19 (8.9)	1 (0.5)	54 (12.3)	2 (0.5)
	Female	175 (16.1)	6 (0.6)	104 (33.1)	2 (0.6)	32 (12.7)	0	39 (7.5)	4 (0.8)
Respiratory rate	All	649 (32.1)	11 (0.5)	256 (42.8)	8 (1.3)	159 (34.2)	2 (0.4)	234 (24.4)	1 (0.1)
	Male	263 (28.1)	7 (0.7)	123 (43.3)	5 (1.8)	62 (29.0)	2 (0.9)	78 (17.8)	0
	Female	386 (35.6)	4 (0.4)	133 (42.4)	3 (1.0)	97 (38.6)	0	156 (30.0)	1 (0.2)
Axillary temperature	All	367 (18.2)	8 (0.4)	137 (22.9)	4 (0.7)	92 (19.8)	3 (0.6)	138 (14.4)	1 (0.1)
	Male	161 (17.2)	4 (0.4)	57 (20.1)	4 (1.4)	39 (18.2)	0	65 (14.8)	0
	Female	206 (19.0)	4 (0.4)	80 (25.5)	0	53 (21.1)	3 (1.2)	73 (14.0)	1 (0.2)
Systolic blood pressure only	All	372 (18.4)	3 (0.1)	130 (21.7)	0	100 (21.5)	0	142 (14.8)	3 (0.3)
	Male	122 (13.0)	3 (0.3)	54 (19.0)	0	36 (16.8)	0	32 (7.3)	3 (0.7)
	Female	250 (23.0)	0	76 (24.2)	0	64 (25.5)	0	110 (21.2)	0
Diastolic blood pressure only	All	218 (10.8)	5 (0.2)	76 (12.7)	1 (0.2)	33 (7.1)	1 (0.2)	109 (11.4)	3 (0.3)
	Male	97 (10.4)	3 (0.3)	33 (11.6)	1 (0.4)	17 (7.9)	1 (0.5)	47 (10.7)	1 (0.2)
	Female	121 (11.2)	2 (0.2)	43 (13.7)	0	16 (6.4)	0	62 (11.9)	2 (0.4)
Both systolic and diastolic	All	178 (8.8)	30 (1.5)	101 (16.9)	1 (0.2)	30 (6.5)	0	47 (4.9)	29 (3.0)
	Male	103 (11.0)	13 (1.4)	55 (19.4)	1 (0.4)	19 (8.9)	0	29 (6.6)	12 (2.7)
	Female	75 (6.9)	17 (1.6)	46 (14.6)	0	11 (4.4)	0	18 (3.5)	17 (3.3)
ECG		*n* = 1,602 (806 male, 796 female)		*n* = 534 (251 male, 283 female)		*n* = 406 (197 male, 209 female)		*n* = 662 (358 male, 304 female)	
	All	444 (27.7)	22 (1.4)	165 (30.9)	2 (0.4)	100 (24.6)	2 (0.5)	179 (27.0)	18 (2.7)
	Male	274 (34.0)	14 (1.7)	82 (32.7)	1 (0.4)	58 (29.4)	2 (1.0)	134 (37.4)	11 (3.1)
	Female	170 (21.4)	8 (1.0)	83 (29.3)	1 (0.4)	42 (20.1)	0	45 (14.8)	7 (2.3)

CS = clinically significant; ECG = electrocardiogram; NCS = not clinically significant. Values are *n* (%). Reference ranges are available in Supplemental Table 7.

**Table 3:** Numbers (%) of adults (BMI) and children (BMI for age or weight for age) with anthropometric parameters outside the reference interval at screening

Parameter (reference interval)		Total		Age Group					
				1.5–9 Years		10–17 Years		18–50 Years	
BMI (18–30 kg/m^2^; *n* = 958)	Total	958		NA		NA		958	
	Male	438		NA		NA		438	
	Female	520		NA		NA		520	
		<LLRi, *N* (%)	>ULRi, *N* (%)	<LLRi, *N* (%)	>ULRi, *N* (%)	<LLRi, *N* (%)	>ULRi, *N* (%)	<LLRi, *N* (%)	>ULRi, *N* (%)
	All	4 (0.4)	89 (9.3)	NA	NA	NA	NA	4 (0.4)	89 (9.3)
	Male	1 (0.2)	16 (3.7)	NA	NA	NA	NA	1 (0.2)	16 (3.7)
	Female	3 (0.6)	73 (14.0)	NA	NA	NA	NA	3 (0.6)	73 (14.0)
Weight for age (1.5–9 years) or BMI for age (10–17 years) (Z-score ± 2 SDs; *n* = 1,063)	Total	1,063		598		465		NA	
	Male	498		284		214		NA	
	Female	565		314		251		NA	
		<LLRi, *N* (%)	>ULRi, *N* (%)	<LLRi, *N* (%)	>ULRi, *N* (%)	<LLRi, *N* (%)	>ULRi, *N* (%)	<LLRi, *N* (%)	>ULRi, *N* (%)
	All	2 (0.2)	12 (1.1)	1 (0.2)	3 (0.5)	1 (0.2)	9 (1.9)	NA	NA
	Male	1 (0.2)	4 (0.8)	1 (0.4)	2 (0.7)	0	2 (0.9)	NA	NA
	Female	1 (0.2)	8 (1.4)	0	1 (0.3)	1 (0.4)	7 (2.8)	NA	NA

BMI = body mass index; LLRi = lower limit of reference interval; NA = not applicable; ULRi = upper limit of reference interval.

### Health data from CRC screening results.

**Figure 4.**
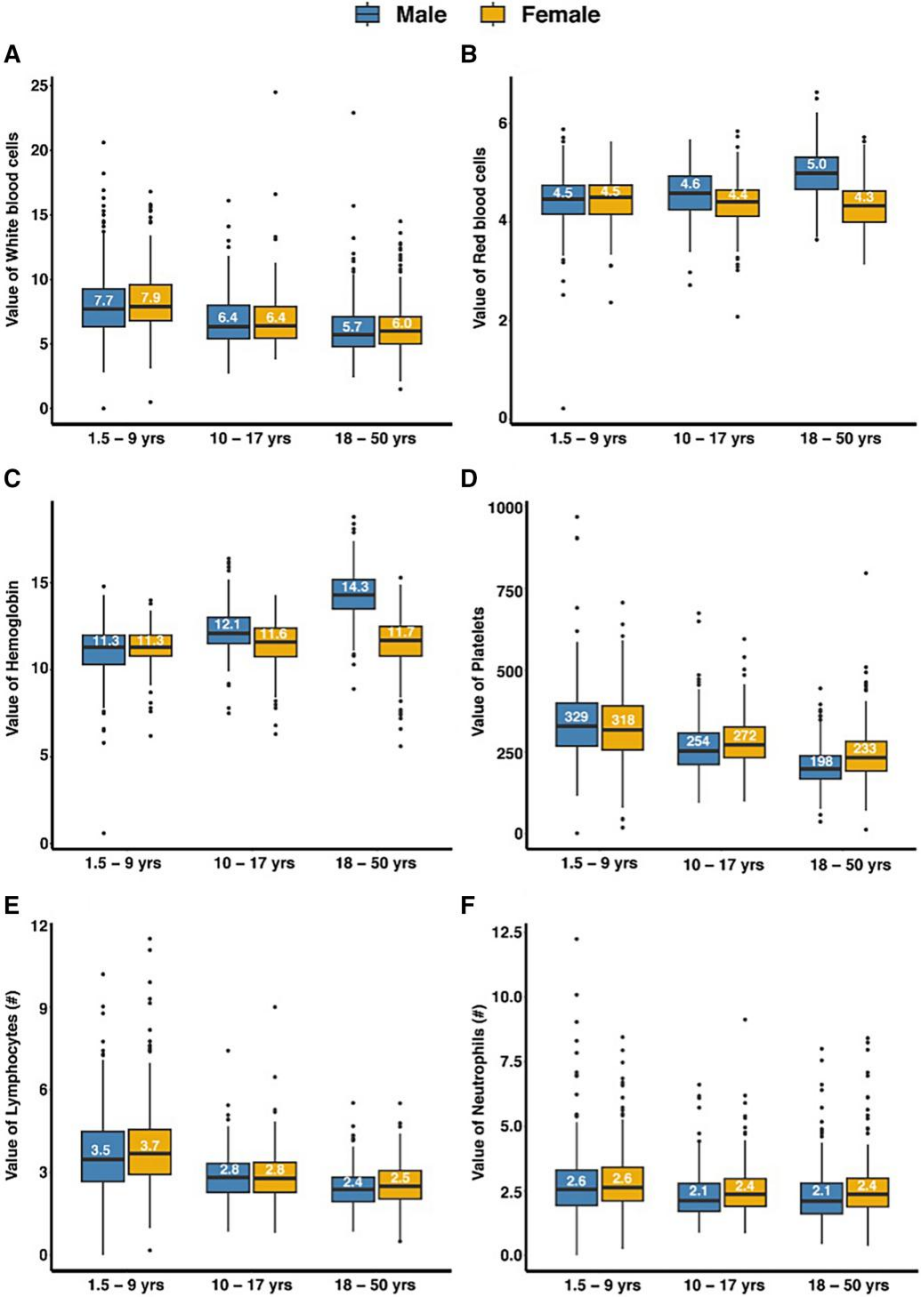

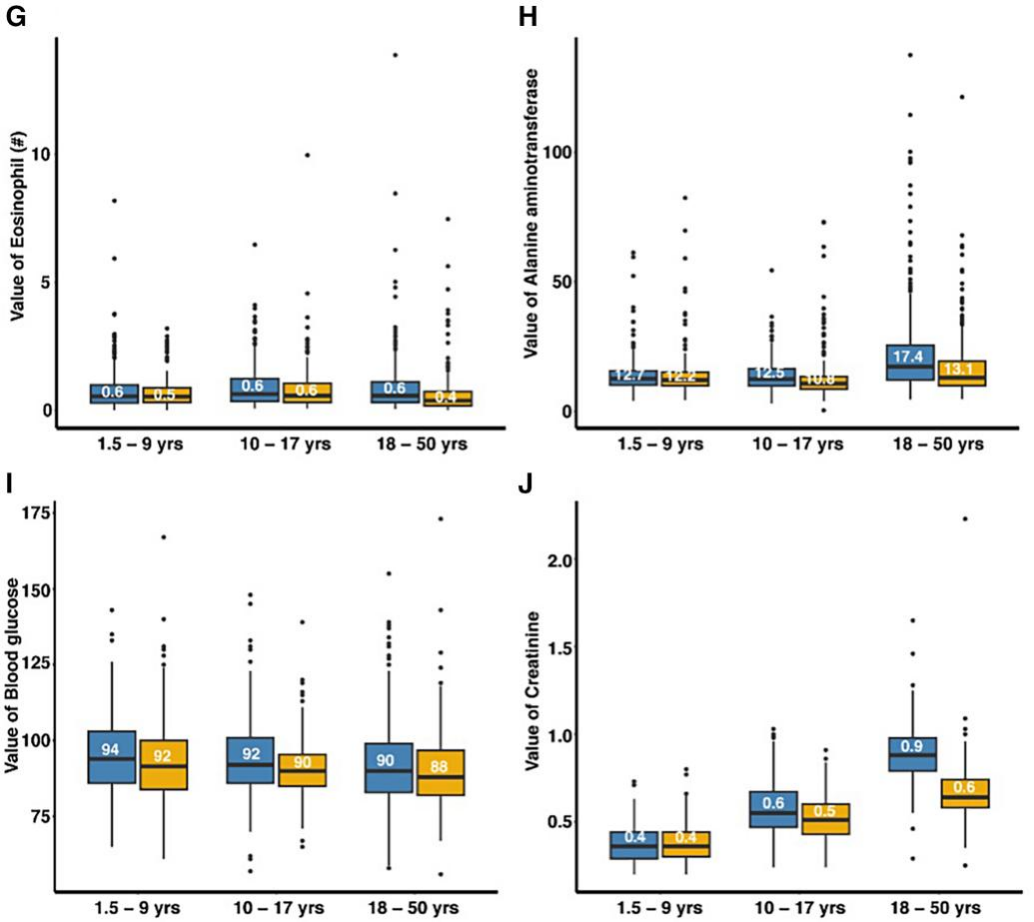
Distributions of laboratory parameters of volunteers who participated in the Equatorial Guinea Pilot Study for Recruitment, Screening and Participant Registry (EGRESPAR), stratified by age and sex. Panels **A–G** show the hematological parameters, and panels **H–J** show the biochemistry parameters. The panels show the distribution of (**A**) white blood cells (10^3^/*µ*L), (**B**) red blood cells (10^6^/*µ*L), (**C**) hemoglobin (g/dL), (**D**) platelets (10^3^/*µ*L), (**E**) lymphocytes (10^3^/*µ*L), (**F**) neutrophils (10^3^/*µ*L), (**G**) eosinophils (10^3^/*µ*L), (**H**) alanine aminotransferase (U/L), (**I**) blood glucose (mg/dL), and (**J**) creatinine (mg/dL). The boxes show the median and the upper and lower quartiles, and the whiskers define maximum and minimum values, excluding outliers.

Among the 1,603 participants who attended the CRC visit, 217 (13.6%) were TBS-positive for malaria, accounting for 11.7%, 18.9%, and 12% of the total tested in the age categories of 1.5–9 years, 10–17 years, and 18–50 years, respectively; 28 (1.8%) were *L. loa*-positive, accounting for 0.4%, 1.2%, and 3.2% of the total tested in the same age categories; 34 (2.1%) were HIV-positive, accounting for 0%, 0.3%, and 5% of the total tested in the same age categories; 77 (4.8%) were hepatitis B surface antigen-positive, accounting for 0.6%, 4.5%, and 8.5% of the total tested in the same age categories; and 8 (0.5%) were hepatitis C antibody-positive, accounting for 0.4%, 0.3%, and 0.8% of the total tested in the same age categories (Table 4). All participants who tested positive for malaria and *L. loa* were treated, and post-treatment tests were performed to confirm negative status and eligibility for inclusion. Individuals who tested positive for HIV and hepatitis were excluded and provided with post-test counseling, as well as referrals to relevant treatment centers for further management. The safety laboratory parameters most frequently found to be outside the reference interval were eosinophils, which were elevated in 365 (22.8%; upper limit of normal: 1.6 × 10^3^/*µ*L [1.5–9 years], 2.1 × 10^3^/*µ*L [10–17 years], 0.78 × 10^3^/*µ*L [18–50 years]), and random blood glucose, which was elevated in 303 (19%; upper limit of normal: 97.3 mg/dL [1.5–9 years], 95.5 mg/dL [10–17 years], 109.8 mg/dL [18–50 years]). Hemoglobin was lower than the reference interval (8.6–13.2 g/dL [1.5–9 years], 9.6–14.1 g/dL [10–17 years], 12–17.4 g/dL [18–50 years males], 9.60–14.10 g/dL [18–50 years females]) in 179 (11.2%) participants, with the greatest frequency occurring in 10–17-year-olds (22%; Table 5). The distribution of laboratory parameters is presented in Figure 4.

**Table 4:** Number (percentage) of individuals with infections among participants screened at clinical research centers

Category		Total	Age Groups		
			1.5–9 Years	10–17 Years	18–50 Years
Total		1,599	533	405	661
Male		806	251	197	358
Female		793	282	208	303
*Plasmodium* total (% of total screened)	All	217 (13.6)	62 (11.6)	76 (18.8)	79 (12.0)
	Male	115 (14.3)	30 (12.0)	36 (18.3)	49 (13.7)
	Female	102 (12.9)	32 (11.3)	40 (19.2)	30 (9.9)
*P. falciparum* only (% of total positive)	All	185 (85.3)	50 (80.6)	64 (84.2)	71 (89.9)
	Male	95 (82.6)	23 (76.7)	31 (86.1)	41 (83.7)
	Female	90 (88.2)	27 (84.4)	33 (82.5)	30 (100)
*P. falciparum + P. malariae* (% of total positive)	All	30 (13.8)	11 (17.7)	11 (14.5)	8 (10.1)
	Male	18 (15.7)	6 (20.0)	4 (11.1)	8 (16.3)
	Female	12 (11.8)	5 (15.6)	7 (17.5)	0
*P. falciparum + P. ovale* (% of total positive)	All	1 (0.5)	0	1 (1.3)	0
	Male	1 (0.9)	0	1 (2.8)	0
	Female	0	0	0	0
*P. falciparum + P. malariae + P. ovale* (% of total positive)	All	1 (0.5)	1 (1.6)	0	0
	Male	1 (0.9)	1 (3.3)	0	0
	Female	0	0	0	0
*Loa loa*	All	28 (1.8)	2 (0.4)	5 (1.2)	21 (3.2)
	Male	18 (2.2)	0	2 (1.0)	16 (4.5)
	Female	10 (1.3)	2 (0.7)	3 (1.4)	5 (1.7)
HIV	All	34 (2.1)	0	1 (0.2)	33 (5)
	Male	3 (0.4)	0	1 (0.5)	2 (0.6)
	Female	31 (3.9)	0	0	31 (10.2)
Hepatitis B	All	77 (4.8)	3 (0.6)	18 (4.4)	56 (8.5)
	Male	49 (6.1)	2 (0.8)	11 (5.6)	36 (10.1)
	Female	28 (3.5)	1 (0.4)	7 (3.4)	20 (6.6)
Hepatitis C	All	8 (0.5)	2 (0.4)	1 (0.2)	5 (0.8)
	Male	4 (0.5)	0	1 (0.5)	3 (0.8)
	Female	4 (0.5)	2 (0.7)	0	2 (0.7)

*P. falciparum* = *Plasmodium falciparum*; *P. malariae* = *Plasmodium malariae*; *P. ovale* = *Plasmodium ovale*. Values are *n* (%).

**Table 5:** Volunteer numbers (%) with hematological and blood biochemistry parameters outside the reference interval at clinical research center screening*

Parameter^†^		Total	Age Groups		
			1.5–9 Years	10–17 Years	18–50 Years
Total tested		1,599	533	405	661
Male		806	251	197	358
Female		793	282	208	303
Hematology					
White blood cells (increased)	All	75 (4.7)	9 (1.7)	29 (7.2)	37 (5.6)
	Male	39 (4.8)	7 (2.8)	14 (7.1)	18 (5.0)
	Female	36 (4.5)	2 (0.7)	15 (7.2)	19 (6.3)
White blood cells (decreased)	All	61 (3.8)	26 (4.9)	11 (2.7)	24 (3.6)
	Male	35 (4.3)	15 (6.0)	8 (4.1)	12 (3.4)
	Female	26 (3.3)	11 (3.9)	3 (1.4)	12 (4.0)
Lymphocytes (increased)	All	152 (9.5)	6 (1.1)	74 (18.3)	72 (10.9)
	Male	72 (8.9)	3 (1.2)	38 (19.3)	31 (8.7)
	Female	80 (10.1)	3 (1.1)	36 (17.3)	41 (13.5)
Lymphocytes (decreased)	All	34 (2.1)	16 (3.0)	8 (2.0)	10 (1.5)
	Male	19 (2.4)	9 (3.6)	6 (3.0)	4 (1.1)
	Female	15 (1.9)	7 (2.5)	2 (1.0)	6 (2.0)
Neutrophils (increased)	All	33 (2.1)	14 (2.6)	7 (1.7)	12 (1.8)
	Male	17 (2.1)	8 (3.2)	4 (2.0)	5 (1.4)
	Female	16 (2.0)	6 (2.1)	3 (1.4)	7 (2.3)
Neutrophils (decreased)	All	215 (13.4)	21 (3.9)	58 (14.3)	136 (20.6)
	Male	139 (17.2)	12 (4.8)	38 (19.3)	89 (24.9)
	Female	76 (9.6)	9 (3.2)	20 (9.6)	47 (15.5)
Eosinophils (increased)	All	386 (24.1)	35 (6.6)	151 (37.4)	200 (30.3)
	Male	233 (28.9)	22 (8.8)	79 (40.3)	132 (36.9)
	Female	153 (19.3)	13 (4.6)	72 (34.6)	68 (22.4)
Eosinophils (decreased)	All	16 (1.0)	16 (3.0)	0	0
	Male	7 (0.9)	7 (2.8)	0	0
	Female	9 (1.1)	9 (3.2)	0	0
Red blood cells (increased)	All	25 (1.6)	1 (0.2)	0	24 (3.6)
	Male	25 (3.1)	1 (0.4)	0	24 (6.7)
	Female	0	0	0	0
Red blood cells (decreased)	All	59 (3.7)	23 (4.3)	17 (4.2)	19 (2.9)
	Male	24 (3.0)	13 (5.2)	8 (4.1)	3 (0.8)
	Female	35 (4.4)	10 (3.5)	9 (4.3)	16 (5.3)
Hemoglobin (decreased)	All	157 (9.8)	21 (3.9)	86 (21.2)	50 (7.6)
	Male	106 (13.2)	15 (6.0)	73 (37.1)	18 (5.0)
	Female	51 (6.4)	6 (2.1)	13 (6.3)	32 (10.6)
Platelets (increased)	All	201 (12.6)	43 (8.1)	97 (24.0)	61 (9.2)
	Male	80 (9.9)	23 (9.2)	40 (20.3)	17 (4.7)
	Female	121 (15.3)	20 (7.1)	57 (27.4)	44 (14.5)
Platelets (decreased)	All	44 (2.8)	6 (1.1)	3 (0.7)	35 (5.3)
	Male	25 (3.1)	1 (0.4)	1 (0.5)	23 (6.4)
	Female	19 (2.4)	5 (1.8)	2 (1.0)	12 (4.0)
Biochemistry					
Alanine aminotransferase (increased)	All	109 (6.8)	95 (17.8)	14 (3.5)	0
	Male	49 (6.1)	44 (17.5)	5 (2.5)	0
	Female	60 (7.6)	51 (18.1)	9 (4.3)	0
Alanine aminotransferase (decreased)	All	61 (3.8)	16 (3.0)	6 (1.5)	39 (5.9)
	Male	36 (4.5)	7 (2.8)	1 (0.5)	28 (7.8)
	Female	25 (3.2)	9 (3.2)	5 (2.4)	11 (3.6)
Creatinine (increased)	All	26 (1.6)	6 (1.1)	1 (0.2)	19 (2.9)
	Male	20 (2.5)	2 (0.8)	0	18 (5.0)
	Female	6 (0.8)	4 (1.4)	1 (0.5)	1 (0.3)
Creatinine (decreased)	All	221 (13.8)	26 (4.9)	158 (39.0)	37 (5.6)
	Male	90 (11.2)	14 (5.6)	73 (37.1)	3 (0.8)
	Female	131 (16.5)	12 (4.3)	85 (40.9)	34 (11.2)
Blood glucose (increased)	All	301 (18.8)	196 (36.8)	48 (11.9)	57 (8.6)
	Male	174 (21.6)	105 (41.8)	30 (15.2)	39 (10.9)
	Female	127 (16.0)	91 (32.3)	18 (8.7)	18 (5.9)
Blood glucose (decreased)	All	16 (1.0)	0	3 (0.7)	13 (2.0)
	Male	13 (1.6)	0	3 (1.5)	10 (2.8)
	Female	3 (0.4)	0	0	3 (1.0)

Values are *n* (%), with percentage calculated using the figures in the column heading (total tested, male and female for the rows below labeled all, male and female, respectively).

* None of the abnormal values were clinically significant.

^†^ Reference ranges available in Supplemental Tables 5 and 6.

### Characterization of participants entered into the EGRESPAR registry.

A total of 1,378 participants were eligible and agreed to be entered into the participant registry (by age category: 512 [1.5–9 years], 369 [10–17 years], and 497 [18–50 years]). The overall mean age ± SD of those entered was 15.9 ± 10.9 years (Table 6). The mean age ± SD for those in the three age categories were 5.9 ± 2.5, 13.4 ± 2.2, and 28.1 ± 8.1 years, respectively. Overall, similar numbers of males and females were enrolled. The percentages of females in the age categories of 1.5–9 years, 10–17 years, and 18–50 years were 53.1%, 51.2%, and 42.7%, respectively. The number of participants from the selected 12 administrative areas of Bioko Island were as follows: Fishtown III Ballares 66, Hacienda la Natividad I 27, Cipriano Tómo 59, San Luis II 57, Vigatana 80, Eulogio Oyo Riquesa 23, Vivienda Sociales detrás de GePetrol 412, Buena Esperanza II 131, Magdalena Mora 35, Sacriba Fang 24, Potao 52, and Basupú 412.

**Table 6:** Demographic characteristics of 1,378 participants in the Equatorial Guinea Pilot Study for Recruitment, Screening and Participant Registry, number (percent)

Parameter		Total	Age Groups		
			1.5–9 Years	10–17 Years	18–50 Years
*N*		1,378	512 (37.2)	371 (26.9)	495 (35.9)
Sex, *n* (%)					
Male		705 (51.2)	240 (46.9)	181 (48.8)	284 (57.4)
Female		673 (48.8)	272 (53.1)	190 (51.2)	211 (42.6)
Age					
Mean ± SD (range), years	All	15.9 ± 10.9 (1.6–50.4)	5.9 ± 2.4 (1.6–10.0)	13.4 ± 2.2 (10.1–17.9)	28.2 ± 8.1 (18.0–50.4)
	Male	16.5 ± 10.6 (1.9–49.7)	6.0 ± 2.3 (1.9–10.0)	13.5 ± 2.2 (10.1–17.9)	27.2 ± 7.7 (18.0–49.7)
	Female	15.4 ± 11.2 (1.6–50.4)	5.9 ± 2.4 (1.6–10.0)	13.2 ± 2.1 (10.0–17.9)	29.4 ± 8.4 (18.0–50.4)
Education (127 lacking data)	All	1,251	386 (30.9)	371 (29.7)	494 (39.5)
	Male	658	194 (29.5)	181 (27.5)	283 (43.0)
	Female	593	192 (32.4)	190 (32.0)	211 (35.6)
Illiterate, *n* (%)	All	3 (0.2)	NA	NA	3 (0.6)
	Male	3 (0.5)	NA	NA	3 (1.1)
	Female	0	NA	NA	0
Primary only, *n* (%)	All	682 (54.5)	386 (100)	261 (70.4)	35 (7.1)
	Male	340 (51.7)	194 (100)	131 (72.4)	15 (5.3)
	Female	342 (57.7)	192 (100)	130 (68.4)	20 (9.5)
Secondary and primary, *n* (%)	All	463 (37.0)	NA	110 (29.6)	353 (71.5)
	Male	244 (37.1)	NA	50 (27.6)	194 (68.6)
	Female	219 (36.9)	NA	60 (31.6)	159 (75.4)
University or higher, *n* (%)	All	103 (8.2)	NA	NA	103 (20.9)
	Male	71 (10.8)	NA	NA	71 (25.1)
	Female	32 (5.4)	NA	NA	32 (15.2)
Residence	All	1,378	512 (37.2)	371 (26.9)	495 (35.9)
	Male	705 (51.2)	240 (46.9)	181 (48.8)	284 (57.4)
	Female	673 (48.8)	272 (53.1)	190 (51.2)	211 (42.6)
Fishtown III Ballares	All	65 (4.7)	21 (4.1)	22 (5.9)	22 (4.4)
	Male	28 (4.0)	7 (2.9)	7 (3.9)	14 (4.9)
	Female	37 (5.5)	14 (5.1)	15 (7.9)	8 (3.8)
Hacienda la Natividad I	All	27 (2.0)	9 (1.8)	4 (1.1)	14 (2.8)
	Male	15 (2.1)	6 (2.5)	1 (0.6)	8 (2.8)
	Female	12 (1.8)	3 (1.1)	3 (1.6)	6 (2.8)
Cipriano Tomo	All	59 (4.3)	22 (4.3)	13 (3.5)	24 (4.8)
	Male	28 (4.0)	7 (2.9)	3 (1.7)	18 (6.3)
	Female	31 (4.6)	15 (5.5)	10 (5.3)	6 (2.8)
San Luis II	All	57 (4.1)	20 (3.9)	13 (3.5)	24 (4.8)
	Male	28 (4.0)	9 (3.8)	5 (2.8)	14 (4.9)
	Female	29 (4.3)	11 (4.0)	8 (4.2)	10 (4.7)
Vigatana	All	80 (5.8)	26 (5.1)	17 (4.6)	37 (7.5)
	Male	46 (6.5)	13 (5.4)	10 (5.5)	23 (8.1)
	Female	34 (5.1)	13 (4.8)	7 (3.7)	14 (6.6)
Eulogio Oyo Riquesa	All	23 (1.7)	5 (1.0)	10 (2.7)	8 (1.6)
	Male	13 (1.8)	2 (0.8)	5 (2.8)	6 (2.1)
	Female	10 (1.5)	3 (1.1)	5 (2.6)	2 (0.9)
Vivienda Sociales detras de GePetrol	All	404 (29.3)	160 (31.3)	97 (26.1)	147 (29.7)
	Male	189 (26.8)	78 (32.5)	45 (24.9)	66 (23.2)
	Female	215 (31.9)	82 (30.1)	52 (27.4)	81 (38.4)
Buena Esperanza II	All	131 (9.5)	46 (9.0)	39 (10.5)	46 (9.3)
	Male	69 (9.8)	19 (7.9)	22 (12.2)	28 (9.9)
	Female	62 (9.2)	27 (9.9)	17 (8.9)	18 (8.5)
Magdalena Mora	All	35 (2.5)	16 (3.1)	13 (3.5)	6 (1.2)
	Male	14 (2.0)	7 (2.9)	3 (1.7)	4 (1.4)
	Female	21 (3.1)	9 (3.3)	10 (5.3)	2 (0.9)
Sacriba Fang	All	24 (1.7)	11 (2.1)	8 (2.2)	5 (1.0)
	Male	10 (1.4)	4 (1.7)	3 (1.7)	3 (1.1)
	Female	14 (2.1)	7 (2.6)	5 (2.6)	2 (0.9)
Potao	All	52 (3.8)	23 (4.5)	16 (4.3)	13 (2.6)
	Male	31 (4.4)	15 (6.3)	11 (6.1)	5 (1.8)
	Female	21 (3.1)	8 (2.9)	5 (2.6)	8 (3.8)
Basupu	All	414 (30.0)	150 (29.3)	118 (31.8)	146 (29.5)
	Male	228 (32.3)	71 (29.6)	65 (35.9)	92 (32.4)
	Female	186 (27.6)	79 (29.0)	53 (27.9)	54 (25.6)
Other places	All	7 (0.5)	3 (0.6)	1 (0.3)	3 (0.6)
	Male	6 (0.9)	2 (0.8)	1 (0.6)	3 (1.1)
	Female	1 (0.1)	1 (0.4)	0	0

Values are *n* (%). NA = not applicable.

### Early termination of the EGRESPAR.

While the study was underway, development priorities shifted because of the creation of a new genetically-attenuated PfSPZ parasite demonstrating a replication-competent, late-liver stage arresting (LARC) phenotype developed by Sanaria and its collaborators at the Seattle Children’s Research Institute.^37^ This new vaccine, *Plasmodium falciparum* sporozoite late-liver stage arresting replication competent vaccine (PfSPZ-LARC2 Vaccine) held the potential for safety and tolerability equal to that of PfSPZ Vaccine, combined with superior efficacy achieved at less than one-quarter the dose, thereby improving the chances of successful malaria elimination and reducing the cost of goods. However, as the vaccine had not yet been clinically tested, this pivot was not compatible with the timelines specified by the BIMEP funders. Additionally, in mid-February 2020, the SARS-CoV-2 pandemic reached Africa, leading to the cessation of household visits and, a month later, to the closure of the study when the clinical team, clinical laboratory, and funding were reassigned by MINSABS to confront the pandemic.

## DISCUSSION

*Plasmodium falciparum* malaria is a persistent health concern on Bioko Island,^3^ mirroring the situation in much of sub-Saharan Africa.^1^ The BIMEP, one of Africa’s most comprehensive and well-funded malaria control programs,^38^ achieved progressive reductions in malaria prevalence during the first 8 years of implementation; however, the impact plateaued, leading to an ambitious plan to develop, license, and field a malaria vaccine chosen for its potential to block infection. Sanaria’s PfSPZ vaccines were selected for development, recognizing that RTS,S/AS01E and R21/Matrix M, now approved for use in infants and young children in several sub-Saharan countries, were not sufficiently efficacious against *P. falciparum* infection to consider their use for malaria elimination^13^ (in the one published trial in which RTS,S/AS01 was assessed in African adults for protection against *P. falciparum* infection, it did not show significant efficacy^39^). Supported by a consortium of funders and collaborators, BIMEP established the long-term goal of supplementing traditional malaria control measures with MVPs to eliminate *P. falciparum* transmission on Bioko Island. Six clinical trials were completed toward this objective, including five assessing the radiation-attenuated PfSPZ Vaccine and one assessing PfSPZ-CVac (CQ). The demonstrations of safety, good tolerability, and protection against CHMI from these trials,^14^^–^^17^^,^^19^^–^^21^ combined with the results of other studies of PfSPZ vaccines in the United States, European Union, and Africa,^22^^–^^30^^,^^40^^–^^44^ led to the planning of a phase 3 trial for licensure. The EGRESPAR was conducted to optimize recruitment practices, assess the health status of the target population for enrollment, and generate a registry of eligible individuals in three age groups. The study used the eligibility criteria planned for the phase 3 trial.

The participants in this study were from areas of higher malaria prevalence in and around Malabo. They considered themselves (or were considered by their parents) to be healthy. Tier 1 screening of 2,021 individuals at the RVs focused on medical history, vital signs, and anthropometric measurements, resulting in 418 (20.7%) exclusions. Clinically relevant abnormalities sufficient for exclusion were evident in the medical history or physical examination, including chronic illnesses and obesity in 187 cases (Tables 2–4). A similar number of women were unwilling to follow the strict pregnancy prevention measures required. The 1,603 (79.3%) individuals who passed the tier 1 assessment were further screened via ECG, physical examination, and laboratory tests at the CRC, including tests for malaria, *L. loa*, HIV, and hepatitis B and C. An additional 225 individuals were excluded, with 118 of these exclusions due to chronic infections. As expected, there were variations in the prevalence of chronic infections among participants in different age categories (Table 4). Except for malaria prevalence, which was highest in adolescents, the prevalence of other conditions was at least two-fold higher among adults compared with children.

The screening parameters used at RVs and for laboratory assessment at the CRC for the present study resulted in higher exclusion rates among adult volunteers than among children. These exclusions reflected the concern that chronic medical conditions could potentially interfere with the interpretation of results and jeopardize the safety of participants selected for the phase 3 vaccine trial.

Overall, a balanced sex ratio was achieved for the registry, with a slightly higher percentage of females among children and a higher percentage of males among adults. The latter is explained by the unwillingness of many women to meet the requirements for avoiding pregnancy and breastfeeding. There was variation in the number of participants from different administrative areas. The highest number of participants were registered from the densely populated semi-rural (Basupú) and urban (Vivienda Sociales de Gepetrol and Buena Esperanza) locations. Other areas were less densely populated, with households dispersed over larger areas. The majority of participants had age-appropriate levels of education (Table 6). The majority of adults indicated either secondary school education (71.6%) or university level (20.8%), with less than 8% indicating primary school only or illiterate.

## CONCLUSION

The EGRESPAR was halted because of changing development priorities and the coronavirus disease 2019 pandemic before reaching the target numbers for the registry. However, it largely achieved its major objectives, including the establishment of a stepwise screening approach that began at the community level (RVs) and progressed to the clinic level (CRC). This approach enabled the study team to optimize the use of limited resources, which is a key consideration for the efficient conduct of a phase 3 trial. The selection, upgrade, and practical use of five satellite sites and the BIMEP CRC created the necessary infrastructure for the administration of investigational products and clinical follow-up during the phase 3 program. Furthermore, the conduct of the EGRESPAR improved knowledge of the disease dynamics among prospective trial participants in the study areas, supported capacity building, and instilled confidence in the implementation teams that conducting a 2,100-person trial was feasible. Despite its interruption and the postponement of further phase 3 planning, the EGRESPAR provided a foundation for the success of any future clinical trial research on Bioko Island.

## Supplemental Materials

10.4269/ajtmh.24-0143 Supplemental Materials
