# Supplementary material for: Assessment of Health Status and Creation of a Registry of Potential Research Participants Aged 1.5 to 50 Years on Bioko Island, Equatorial Guinea
Source: Am J Trop Med Hyg. 2025 Apr 8;112(6):1364–77. doi: 10.4269/ajtmh.24-0143 (PMC12139550; doi:10.4269/ajtmh.24-0143)
Supplement: Supplemental Materials [file tpmd240143.SD1.pdf]

Supplemental Material for:

Assessment of Health Status and Creation of a Registry of Potential Research Participants Age 1.5 to 50 Years on Bioko Island, Equatorial Guinea

López Mikue, MSA et al.

Supplemental Table 1. Inclusion / Exclusion Criteria

Inclusion Criteria for Acceptance into Clinical Trials Registry [point where criterion applied (either Household/pre-consent, Recruitment Venue, or Clinical Research Center), and classification into categories “clinical” or “compliance” at Recruitment Venue]
1. Age 1.5-50 years at time of anticipated future consent/assent. [Household & Pre-consent]
2. Males and non-pregnant, non-lactating females in general good health. [Household & Pre-consent]
3. Age-appropriate understanding and communication in Spanish for participant or parent/guardian. [Household & Pre-consent]
4. Currently residing in the selected community and willing and able to attend the required appointments at the CRC for the next two years. [Household & Pre-consent]
5. Agree to be contacted by telephone and by home visit. [Household & Pre-consent]
6. Demonstrate understanding of the study by responding correctly to 10 out of 10 true/false statements about the study. (for those who fail to respond correctly to all true/false statements in their first attempt a second and final attempt will be granted). [Recruitment Venue - Compliance]
7. Agree (if eligible), to be registered in the database of potential participants for upcoming clinical trial. [Recruitment Venue - Compliance]
8. Willing to consider providing consent on pregnancy prevention and breast feeding using highly effective and documented birth control method applicable locally [Recruitment Venue - Compliance]
9. Agree to release medical information and inform a study doctor about contraindications for participation in the future studies. [Recruitment Venue - Compliance]
10. Agree to be attended to by study doctors and take all medications prescribed during the study period. [Recruitment Venue - Compliance]
11. Willing not to take any prescription or herbal medicine obtained outside of the study without first notifying the study team during future studies. [Recruitment Venue - Compliance]
12. Agree to provide contact information of a third-party household member and/or close friend to the study team. [Recruitment Venue - Compliance]
13. Willing not to participate in another clinical trial during the study period or donate blood during the future study period. [Recruitment Venue - Compliance]
14. Agree to undergo HIV, hepatitis B (HBV) and hepatitis C (HCV) testing and all other investigations as stipulated in the protocols or needed for clinical reasons by the investigators. [Recruitment Venue - Compliance]
15. Willing to consider not to take drugs with antimalarial activity during the future trials. [Recruitment Venue - Compliance]
16. Body Mass Index (BMI) 18-30, growth assessment within +/- 2SD for age. [Recruitment Venue - Clinical]
17. Sufficiently healthy as per the clinical judgement based on medical history, examination and investigations done in this study. [Recruitment Venue - Clinical] & [CRC]

Exclusion Criteria for Acceptance into Clinical Trials Registry [point where criterion applied (either Household/pre-consent, Recruitment Venue, or Clinical Research Center), and classification into categories "clinical" or "compliance" at Recruitment Venue]
1. Previous receipt of an investigational malaria vaccine or drug in the last 5 years. [Household & Pre-consent]
2. Two persons of the same age group are already enrolled from the same household (a maximum of two individuals for each age group may be enrolled in the participants registry from the same household. Participants who are withdrawn or voluntarily withdraw from the registry after enrollment, may also be replaced). [Household & Pre-consent]
3. Regular or planned use of immunoglobulin and/or any blood products in the next 2 years. [Recruitment Venue - Clinical]
4. History of drug or alcohol use that meets criteria for "substance abuse" as defined by the WHO (*https://www.who.int/topics/substance_abuse/en/*). [Recruitment Venue - Clinical]
5. Known allergic reactions to components of PfSPZ Vaccine (sporozoites, phosphate buffered saline, human serum albumen), normal saline, or artemether/lumefantrine (AL). [Recruitment Venue - Clinical]
6. History of anaphylaxis or other life-threatening reaction to a vaccine or drug. [Recruitment Venue - Clinical]
7. History of arrhythmias, prolonged QT-interval or other cardiac disease, or clinically significant abnormalities on electrocardiogram (ECG) at screening. [Recruitment Venue - Clinical]
8. History of non-febrile seizures or complex febrile seizures. [Recruitment Venue - Clinical]
9. History of chronic illness including diabetes mellitus, cancer, HIV/AIDS, tuberculosis, and sickle cell anemia. [Recruitment Venue - Clinical]
10. History of clinically significant developmental delay as evaluated by the investigator. [Recruitment Venue - Clinical]
11. Any clinically significant deviation from the normal range in biochemistry or hematology tests measured at screening and not resolving.[CRC]
12. HIV, hepatitis B virus or hepatitis C virus tests indicating ongoing infection. [CRC]
13. Positive urine or blood pregnancy test in females of child-bearing potential (9 years old or older at the time of enrollment).[CRC]
14. Signs and symptoms of tuberculosis (e.g., chronic cough, night sweats, chronic fever, enlarged lymph nodes, unintended weight loss).[CRC]
15. Medical history, symptoms, signs and/or laboratory values suggestive of a systemic disorder or chronic illness (including renal, hepatic, blood, cardiovascular, pulmonary, skin, immunodeficiency, psychiatric, and other conditions) which could interfere with the interpretation of the study results or compromise the health of the research participant. [Recruitment Venue - Clinical] & [CRC]
16. Any medical, psychiatric, social, behavioral or occupational condition or situation that, in the judgment of the principal investigator, impairs a research subject's ability to give informed consent, increases the risk to the research subject of participation in the future studies, affects the

ability of the research subject to participate fully in the study, or might negatively impact the quality, consistency, integrity or interpretation of data derived from their participation in the future study. [Recruitment Venue - Clinical] & [CRC]

Supplemental Table 2. Criteria associated with failure at household level

	All* (n=4156)	Age groups in years		
		1.5 – 9 (n=1130)	10 – 17 (n=986)	18 – 50 (n=2040)
Did not understand Spanish	139 (3.3)	10 (0.9)	6 (0.6)	123 (6.0)
Plan to stay out of the study area	219 (5.3)	30 (2.7)	39 (4.0)	150 (7.4)
Did not agree to attend screening	156 (3.7)	47 (4.2)	35 (3.5)	74 (3.6)
Received investigational product	4 (0.1)	1 (0.1)	1 (0.1)	2 (0.1)
Totals	518	88	81	349

*A house can have more than one participant providing a reason for failure

Supplemental Table 3. Criteria associated with exclusion of screened participants at recruitment venue (RV) level (n=418)

		Total	Age groups in years		
		(418)	1.5 - 9	10 - 17	18 - 50
Failure to both, clinical and non-clinical criteria at RV	All	38 of 418	1	3	34
	Male	3	1	0	2
	Female	35	0	3	32
Failure to at least 1 non-clinical criteria at RV	All	235	44	38	153
	Male	55	20	9	26
	Female	180	24	29	127
Listing* and occurrence of non-clinical criteria associated with failure at RV level					
Not in age 1.5-50 years at time of anticipated future consent/assent.	All	1	0	0	1
	Male	1	0	0	1
	Female	0	0	0	0
Males and non-pregnant, non-lactating females who were not in general good health.	All	5	0	0	5
	Male	0	0	0	0
	Female	5	0	0	5
Currently not residing in the selected community and not willing and not able to attend the required appointments at the CRC for the next two years.	All	55	16	8	31
	Male	29	7	4	18
	Female	26	9	4	13
Did not agree to be contacted by telephone and by home visit	All	61	28	14	19
	Male	25	13	5	7
	Female	36	15	9	12
Did not agree to be in EGRESPAR registry	All	2	0	0	2
	Male	0	0	0	0
	Female	2	0	0	2
Unwilling to provide consent on pregnancy prevention and breast feeding	All	110	NA	16	94
	Male	NA	NA	NA	
	Female	110	NA	16	94
Unwilling not to take any prescription or herbal medicine obtained outside of the study	All	2	0	0	0
	Male	0	0	0	0
	Female	2	0	0	2
Did not agree to undergo HIV, hepatitis B (HBV) and hepatitis C (HCV) tests and all other investigations	All	3	0	0	3
	Male	0	0	0	0
	Female	3	0	0	3
Failure to at least 1 clinical criterion at RV	All	202	20	20	162
	Male	66	13	6	47
	Female	136	7	14	115
Listing* and occurrence of clinical criteria associated with failure at RV					
Anthropometric parameters outside reference interval	All	107	4	10	93
	Male	22	3	2	17
	Female	85	1	8	76
	All	101	10	11	80
	Male	41	6	5	30

Not sufficiently healthy as per the clinical judgement based on medical history, examination and investigations done in this study	Female	60	4	6	50
History of drug or alcohol use that meets criteria for "substance abuse"	All	9	0	0	9
	Male	6	0	0	6
	Female	3	0	0	3
History of non-febrile seizures or complex febrile seizures	All	7	0	2	5
	Male	5	0	2	3
	Female	2	0	0	2
History of chronic illness including diabetes mellitus, cancer, HIV/AIDS, tuberculosis, and sickle cell anemia	All	49	8	5	36
	Male	19	5	2	12
	Female	30	3	3	24
History of clinically significant developmental delay as evaluated by the investigator.	All	10	1	5	4
	Male	5	1	2	2
	Female	5	0	3	2
Medical history, symptoms, signs and/or laboratory values suggestive of a systemic disorder or chronic illness	All	104	15	11	78
	Male	48	10	6	32
	Female	56	5	5	46
Any medical, psychiatric, social, behavioral, or occupational condition	All	71	6	9	56
	Male	33	4	5	24
	Female	38	2	4	32

One person can have more than one exclusion criteria
** See full list at Supplemental Table 1*

Supplemental Table 4. Criteria associated with failure at CRC level

		Total (225)	Age groups in years		
			1.5 - 9	10 - 17	18 - 50
**Failure to at least 1 non-clinical criterion at CRC**	All	8	2	2	4
	Male	3	1	1	1
	Female	5	1	1	3
Listing* and occurrence of non-clinical criteria associated with failure at CRC level					
Currently residing in the selected community and willing and able to attend the required appointments at the CRC for the next two years	All	1	1	0	0
	Male	0	0	0	0
	Female	1	1	0	0
Demonstrate understanding of the study by responding correctly to 10 out of 10 true/false statements about the study. (for those who fail to respond correctly to all true/false statements in their first attempt a second and final attempt will be granted)	All	1	1	0	0
	Male	1	1	0	0
	Female	0	0	0	0
Agree (if eligible), to be registered in the database of potential participants for upcoming clinical trial	All	1	0	1	0
	Male	0	0	0	0
	Female	1	0	1	0
Willing to consider providing consent on pregnancy prevention and breast feeding using highly effective and documented birth control method applicable locally	All	1	0	0	1
	Male	0	0	0	0
	Female	1	0	0	1
History of arrhythmias, prolonged QT-interval or other cardiac disease, or clinically significant abnormalities on electrocardiogram (ECG) at screening	All	3	0	1	2
	Male	2	0	1	1
	Female	1	0	0	1
History of chronic illness including diabetes mellitus, cancer, HIV/AIDS, tuberculosis, and sickle cell anemia	All	1	0	0	1
	Male	0	0	0	0
	Female	1	0	0	1
**Failure to at least 1 clinical criterion at CRC**	All	217	20	34	163
	Male	99	10	16	73
	Female	118	10	18	90
Listing* and occurrence of clinical criteria associated with failure at CRC level					
History of arrhythmias, prolonged QT-interval, or other cardiac disease	All	30	2	2	26
	Male	19	1	2	16
	Female	11	1	0	10
Any clinically significant deviation from the safety data reference interval**	All	29	5	5	19
	Male	11	2	1	8
	Female	18	3	4	11
	All	118	5	20	93

Positive HIV, hepatitis B virus or hepatitis C virus tests	Male	55	2	12	41
	Female	63	3	8	52
	All	11	NA	0	11
Positive urine or blood pregnancy test	Male	NA	NA	NA	NA
	Female	11	0	0	11
	All	1	0	1	0
Signs and symptoms of tuberculosis	Male	0	0	0	0
	Female	1	0	1	0
Medical history, symptoms, signs and/or laboratory values suggestive of a systemic disorder or chronic illness	All	180	13	29	138
	Male	83	5	14	64
	Female	97	8	15	74
	All	189	16	29	144
Any medical, psychiatric, social, behavioral or occupational condition	Male	88	8	13	67
	Female	101	8	16	77

One person can have more than one exclusion criteria

** See full list at Supplemental Table 1*

*** These 29 clinically significant deviations from reference intervals were for vital signs, ECG screening, infection screening etc., but not from laboratory abnormalities.*

Supplemental Table 5. Hematology and Chemistry Reference Intervals and Toxicity Grading (Children 6 months to < 11 years)

Reference Intervals: AGE RANGE [Children 6 months to <11 years]							
**HEMATOLOGY**	**6 Months to <6 Years**			**6 Years to <11 Years**			
**WBC** (Leucocyte count)	[5.1-16.2]	10^3 / µl		[4.5-12.9]	10^3 / µl		
**LYMPH** (Lymphocytes)	[2.1-9.5]	10^3 / µl	(30.2-73.2) %	[1.8-6.4]	10^3 / µl	(30.8-64.9) %	
**MONO** (Monocytes)	[0.4-1.6]	10^3 / µl	(5.1-16.8) %	[0.3-1.1]	10^3 / µl	(5.0-14.3) %	
**NEUT** (Neutrophils)	[1.3-6.9]	10^3 / µl	(15.8-57.6) %	[1.2-6.2]	10^3 / µl	(21.3-59.4) %	
**EO** (Eosinophils)	[0.1-1.6]	10^3 / µl	(0.7-17) %	[0.1-2.1]	10^3 / µl	(1.0-25.9) %	
**BASO** (Basophils)	[0.01-0.1]	10^3 / µl	(0.1-1.2) %	[0.01-0.1]	10^3 / µl	(0.1-1.0) %	
**RBC** (Erythrocyte count)	[3.8-5.8]	10^6 / µl		[3.6-5.8]	10^6 / µl		
**HGB** (Hemoglobin)	[8.6-13.2]	g/dL		[9.6-14.1]	g/dL		
**HCT** (Hematocrit)	[27.5-38.9]	%		[28.7-42.3]	%		
**MCV** (Mean corpuscular volume)	[56.4-83.1]	fL		[61.2-85.1]	fL		
**MCH** (Mean corpuscular hemoglobin)	[17.5-28.5]	pg/cell		[20.3-29.2]	pg/cell		
**MCHC** (Mean corpuscular hemoglobin concentration)	[30.1-35.6]	g/dL		[31.3-36.8]	g/dL		
**RDW_SD** (Red-cell distribution width Standard Deviation)	[32.8-48.5]	fL		[32.5-44.5]	fL		
**PLT** (Platelets)	[91-491]	10^3 / µl		[91-456]	10^3 / µl		
**MPV** (Mean Platelet Volume)	[8.9-13.5]	fL		[8.8-14.0]	fL		
**RDW_CV** (Red-cell distribution width Coefficient of Variation)	[12.3-22.5]	%		[12.0-19.4]	%		
**PDW** (Platelet distribution width)	[10.1-21.1]	fL		[10.2-23.3]	fL		
**P-LCR** (Platelet Large Cell Ratio)	[18.0-51.7]	%		[16.8-55.1]	%		
**PCT** (Plateletcrit)	[0.15-0.52]	%		[0.13-0.45]	%		

**BIOCHEMISTRY**	6 Month to <12 Months	1 Year to <6 Years	6 Years to <11 Years
**Alanine aminotransferase (ALT/SGPT)**	[9-33] U/L	[10-28] U/L	[9-35] U/L
**Aspartate aminotransferase (AST/SGOT)**	[26-65] U/L	[27-55] U/L	[21-51] U/L

**Total Bilirubin (TBIL)**	[0.12-1.1] mg/dL	[0.12-0.53] mg/dL	[0.12-0.64] mg/dL
**Creatinine**	[0.16—0.37] mg/dL	[0.2-0.66] mg/dL	[0.31-0.64] mg/dL
**Glucose**	[52.3 -99.1] mg/dL	[54.1-97.3] mg/dL	[48.6-95.5] mg/dL
**Albumin**	[36-48] g/L	[40-49] g/L	[40-48] g/L
**Alkaline Phosphatase**	[164-589] U/L	[153-410] U/L	[174-460] U/L
**γ- Glutamyltransferase(GGT)**	[3.0-34.0] U/L	[3.0-34.0] U/L	[7.0-31.0] U/L
**Bilirubin Direct**	[0-1.1] mg/dL	[0-0.53] mg/dL	[0.03-0.23] mg/dL
**Lactate Dehydrogenase (LDH)**	[360-995] U/L	[360-995] U/L	[277-823] U/L
**Cholesterol**	[2.1-5.7] mmo/L	[2.5-5.3] mmo/L	[2.1-4.9] mmo/L
**Creatinine Kinase**	[45-227] U/L	[73-248] U/L	[83-294] U/L
**Blood Urea Nitrogen (BUN)**	[0.7-3.8] mmo/L	[1.3-4.2] mmo/L	[1.4-4.4] mmo/L
**Potassium**	[4.2-6.5] mmo/L	[3.8-5.9] mmo/L	[3.2-5.2] mmo/L
**Sodium**	[133-139] mmo/L	[133-141] mmo/L	[134-141] mmo/L
**Chloride**	[100-107] mmo/L	[100-108] mmo/L	[98-108] mmo/L
**Calcium**	[2.4-2.8] mmo/L	[2.2-2.7] mmo/L	[2.2-2.6] mmo/L
**Magnesium**	[0.8-1.1] mmo/L	[0.8-1.0] mmo/L	[0.8-1.0] mmo/L

**HEMATOLOGY: Toxicity Grading; (Age Range: 6 months to <11 years)**								
**Parameters**	**Age Group**	**Normal Range**			**Toxicity Grades**			
		**Unit**	**LLN**	**ULN**	**Mild (Grade 1)**	**Moderate (Grade 2)**	**Severe (Grade 3)**	**Potentially life Threatening (Grade 4)**
**HGB – Hemoglobin**	**6mo- <6Y**	g/dL	8.6	13.2	**8- <8.6**	**7-<8**	**<7**	**Cardiac Failure secondary to anemia**
	**6y - <11y**	g/dL	9.6	14.1	**8- <9.6**			
**PLT – Platelets**	**6mo- <6Y**	$10^3/\mu l$	91	491	**75 - <91**	**50 - <75**	**25 - <50**	**<25**
	**6y - <11y**	$10^3/\mu l$	91	456				
**WBC – White Blood-cell Count Low**	**6mo- <6Y**	$10^3/\mu l$	5.1	16.2	**2 - <5.1**	**1.5 - <2**	**1 - <1.5**	**<1**
	**6y - <11y**	$10^3/\mu l$	4.5	12.9	**2 - <4.5**			
**Neutrophils**	**6mo- <6Y**	$10^3/\mu l$	1.3	6.9	**0.8 - <1.3**	**0.6 - <.8**	**0.4 - <.6**	**<0.4**
	**6y - <11y**	$10^3/\mu l$	1.2	6.2	**0.8 - <1.2**			

**Absolute Neutrophils Count Low**	**6mo-12mo**	10³/µl	2.1	9.5	**0.6 - <2.1**	**0.5 - <0.6**	**0.35 - <0.5**	**<0.35**
	**6y - <11y**	10³/µl	1.8	6.4	**0.6 - <1.8**			
**Lymphocytes**	**6mo-12mo**	10³/µl	0.1	1.6	**>1.6 - 2.4**	**>2.4 - 4.8**	**> 4.8**	**Hypereosinophilia**
	**6y - <11y**	10³/µl	0.1	2.1	**>2.1-3.1**	**>3.2 - 6.3**	**> 6.3**	
**BIOCHEMISTRY: Toxicity Grading; (Age Range: 6 months to <11 years)**								
**Parameters**	**Age Group**	**Normal Range**			**Toxicity Grades**			
		**Unit**	**LLN**	**ULN**	**Mild (Grade 1)**	**Moderate (Grade 2)**	**Severe (Grade 3)**	**Potentially life Threatening (Grade 4)**
**Alanine aminotransferase (ALAT)**	**6mo- <12mo**	U/L	9	33	**>33 - 66**	**>66 - 99**	**>99 - 264**	**> 264**
	**1y - <6y**	U/L	10	28	**>28 - 56**	**>56 - 84**	**>84 - 224**	**> 224**
	**6y - <11y**	U/L	9	35	**>35 - 70**	**>70 - 105**	**>105 - 280**	**> 280**
**Aspartate aminotransferase (ASAT)**	**6mo- <12mo**	U/L	26	65	**>65 - 130**	**>130 - 195**	**>195 - 520**	**> 520**
	**1y - <6y**	U/L	27	55	**>55 - 110**	**>110 - 165**	**>165 - 440**	**> 440**
	**6y - <11y**	U/L	21	51	**>51 - 102**	**>102 - 153**	**>153 - 408**	**> 408**
**Total Bilirubin (TBIL) - when accompanied by any increase in other Liver function test**	**6mo - <12mo**	mg/dL	0.12	1.11	**>1.11 - 1.38**	**>1.38-1.65**	**>1.65- 1.94**	**> 1.94**
	**1y - <6y**	mg/dL	0.12	0.53	**>0.53- 0.65**	**>0.65 -0.78**	**>0.78- 0.92**	**> 0.92**
	**6Y - <11y**	mg/dL	0.12	0.64	**>0.64- 0.79**	**>0.79- 0.95**	**>0.95- 1.13**	**> 1.13**
**Total Bilirubin (TBIL) - when Liver function are normal range**	**6mo-12mo**	mg/dL	0	0.11	**>0.11 – 1.66**	**>1.66 – 2.21**	**>2.21- 3.33**	**> 3.33**
	**1y - 5y**	mg/dL	0	0.52	**>0.52 – 0.78**	**>0.78 -1.04**	**>1.04 – 1.58**	**> 1.58**
	**6y - <11y**	mg/dL	0	0.64	**>0.64- 0.95**	**>0.95-1.27**	**>1.27 – 1.93**	**> 1.93**
**CREATININE**	**6mo-12mo**	mg/dL	0.16	0.32	**>0.32 - 0.80**	**>0.80 - 1.10**	**>1.10 – 1.50**	**> 1.50**
	**1y - <2y**	mg/dL	0.2	0.57	**>0.57 – 0.80**	**>0.80 – 1.10**	**>1.10 – 1.50**	**>1.50**
	**2y - <6y**	mg/dL	0.17	0.57	**>0.57-1.0**	**>1.0 – 1.60**	**>1.60 – 2.0**	**> 2.0**
	**6y - <11y**	mg/dL	0.27	0.55	**>0.55 – 1.0**	**>1.0 – 1.60**	**>1.60 – 2.0**	**>2.0**

Supplemental Table 6. Hematology and Chemistry Reference Intervals and Toxicity Grading (Children 11 to Adults 65 years)

Reference Intervals: AGE RANGE [11 years to 65 years]				
**HEMATOLOGY**				
**WBC** (Leucocyte count)	[3.65-9.7]	10^3 / µl		
**LYMPH** (Lymphocytes)	[1.19 - 3.4]	10^3 / µl		
**MONO** (Monocytes)	[0.25-1.41]	10^3 / µl (0 – 13.96) %		
**NEUT** (Neutrophils)	[1.61-5.69]	10^3 / µl (27.9-69.86) %		
**EO** (Eosinophils)	[0 –0.78]	10^3 / µl (0 – 14.15) %		
**BASO** (Basophils)	[0 – 0.05]	10^3 / µl (0 – 0.9) %		
**RBC** (Erythrocyte count)	[3.8-5.67	] 10^6 / µl Male	[3.65-5.84	] 10^6 / µl Female
**HGB**(Hemoglobin)	[12-17.4	] g/dL Male	[9.60-14.10	] g/dL Female
**HCT** (Hematocrit)	[32.8-46.7	] % Male	[28.72-42.30	] % Female
**MCV** (Mean corpuscular volume)	[74.4-92.6]	fL		
**MCH** (Mean corpuscular hemoglobin)	[27.6-34.6]	pg/cell		
**MCHC** (Mean corpuscular hemoglobin concentration)	[35.7-38.8]	g/dL		
**RDW_SD** (Red-cell distribution width Standard Deviation)	[34.97- 49.22]	fL		
**PLT** (Platelets)	[124-312]	10^3 / µl		
**MPV** (Mean Platelet Volume)	[8.3 -13.4]	fL		
**RDW_CV** (Red-cell distribution width Coefficient of Variation)	[11.7- 16.1]	%		
**PDW** (Platelet distribution width)	[9 – 19.14]	fL		
**P-LCR** (Platelet Large Cell Ratio)	[11.17 -51.27]	%		
**PCT** (Plateletcrit)	[0 – 0.36]	%		
**BIOCHEMISTRY**				
**Alanine aminotransferase (ALT/SGPT)**	[0 – 45]	U/L		
**Aspartate aminotransferase (AST/SGOT)**	[15.2 – 58.7]	U/L		
**Total Bilirubin (TBIL)**	[0 – 2.62]	mg/dL		
**Creatinine**	[0.54—1.11]	mg/dL		
**Glucose**	[70.3-109.8]	mg/dL		
**Albumin**	[42.7 – 60]	g/L		
**Alkaline Phosphatase**	[45 – 186.9]	U/L		
**γ- Glutamyltransferase(GGT)**	[8.3 – 108.1]	U/L		
**Bilirubin Direct**	[0 – 0.36]	mg/dL		
**Lactate Dehydrogenase (LDH)**	[127 – 264]	U/L		
**Cholesterol**	[96.7—200.7]	mg/dL		
**Creatinine Kinase**	[77 – 787]	U/L		
**Blood Urea Nitrogen (BUN)**	[7—19]	mg/dL		
**Potassium**	[3.5 – 5]	mmo/L		
**Sodium**	[134 – 142]	mmo/L		
**Chloride**	[97 – 107]	mmo/L		
**Calcium**[2]	[1.99 – 2.52]	mmo/L		

**Magnesium[2]**	[0.67 – 0.97] mmo/L

**HEMATOLOGY: Toxicity Grading; (Age Range: 11-65 YEARS OLD - Healthy)**							
**Parameters**	**Normal Range**			**Toxicity Grades**			
	**Unit**	**LLN**	**ULN**	**Mild (Grade 1)**	**Moderate (Grade 2)**	**Severe (Grade 3)**	**Potentially life Threatening (Grade 4)**
**Leukocyte (WBC) Increase**	$x10^3$/μL	3.65	9.7	**>9.7 - 15**	**>15 - 20**	**>20 - 25**	**> 25**
**Leukocyte (WBC) Decrease**	$x10^3$/μL	3.65	9.7	**<3.65 - 2.5**	**<2.5 - 1.5**	**<1.5 - 1**	**< 1**
**Lymphocytes Decrease**	$x10^3$/μL	1.19	3.4	**<1.19 - 0.75**	**<0.75 - 0.5**	**< 0.5 - 0.25**	**< 0.25**
**Neutrophils Decrease**	$x10^3$/μL	1.61	5.69	**<1.61 - 0.9**	**< 0.9 - 0.6**	**< 0.6 - 0.4**	**<0.4**
**Eosinophils**	$x10^3$/μL	0	0.78	**> 0.78 - 1.5**	**>1.5 - 5**	**> 5**	**Hyper-eosinophilic**
**Hemoglobin (Male)**	g/dL	12	17.4	**<12-8.0**	**<8.0 – 7.0**	**<7.0 – 4.0**	**<4.0**
**Hemoglobin (Female)**	g/dL	9.6	14.1	**<9.6 – 8.0**	**<8.0 – 7.0**	**<7.0 – 4.0**	**<4.0**
**Platelets Decreased**	$x10^3$/μL	124	312	**75 - <124**	**50 - <75**	**25 – <50**	**< 25**

**BIOCHEMISTRY: Toxicity Grading; (Age Range: 11-65 YEARS OLD - Healthy)**								
**Parameters**	**Normal Range**			**Toxicity Grades**				
	**Unit**	**LLN**	**ULN**	**Mild (Grade 1)**	**Moderate (Grade 2)**	**Severe (Grade 3)**	**Potentially life Threatening (Grade 4)**	[illegible]
**Alanine aminotransferase (ALAT)**	U/L	0	45	**>45 – 106.5**	**>106.5 – 209**	**>209 - 410**	**>410**	
**Aspartate aminotransferase (ASAT)**	U/L	15.2	58.7	**>58.7-138.8**	**>138.8-272.3**	**>272.3-534**	**>534**	
**Total bilirubin (TBIL) - when accompanied by any increase in other liver function test**	mg/dL	0	2.62	**> 1.82 - 2.27**	**>2.27 – 2.73**	**>2.73 – 3.18**	**> 3.18**	
**Total bilirubin (TBIL) - when liver function tests are normal range**	mg/dL	0	2.62	**>2.62-3.01**	**>3.01-3.61**	**>3.612-4.19**	**>4.19**	
**Creatinine**	mg/dL	0.54	1.11	**>1.11-1.70**	**>1.70-2.0**	**>2.0-2.5**	**> 2.5 or require dialysis**	

Supplemental Table 7: Vital Signs Reference Intervals

Parameters	Reference Range			
	1 year to < 3 years	3 years to < 6 years	6 years to < 12 years	≥ 12 years
Heart Rate (beats/min)	70-110	65-110	60-95	56-96
Respiratory Rate (breaths/min)	20-30	20-25	14-22	12-20
Temperature (axillary) (ºC)	> 36 ºC – 37.5 ºC			
Systolic Blood Pressure (mmHg)	90-105	95-110	100-120	105-136
Diastolic Blood Pressure (mmHg)	55-70	60-75	60-75	58-84

Supplemental Figure 1. Z-score BMI for age (children 11-17 years)

Reference Charts Adopted from the WHO Growth References: http://www.who.int/growthref/en/

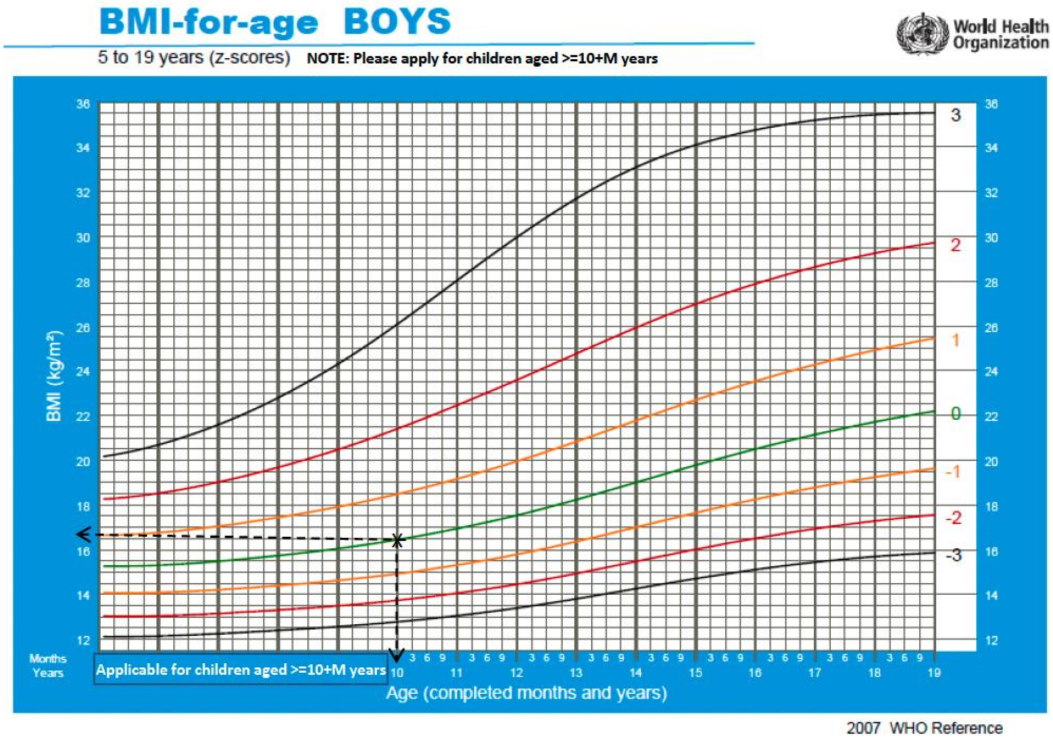

Reference Charts Adopted from the WHO Growth References: http://www.who.int/growthref/en/

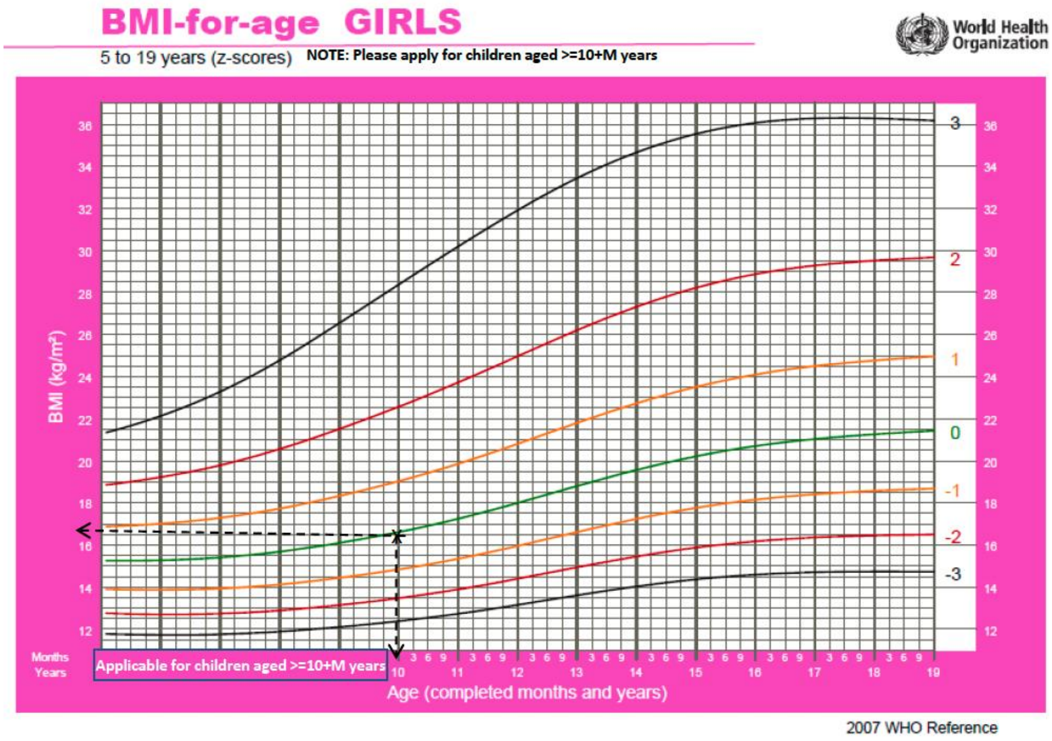

Supplemental Figure 2. Z-score weight for age (children <11 years)

Reference Charts Adopted from the WHO Growth References: http://www.who.int/growthref/en/

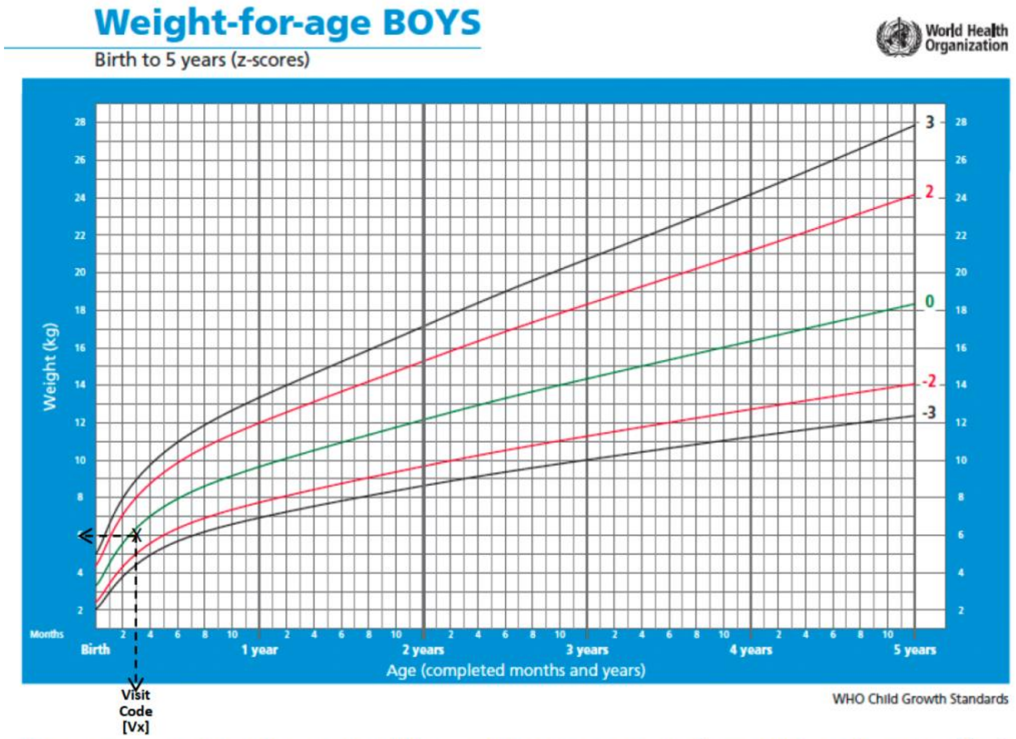

Reference Charts Adopted from the WHO Growth References: http://www.who.int/growthref/en/

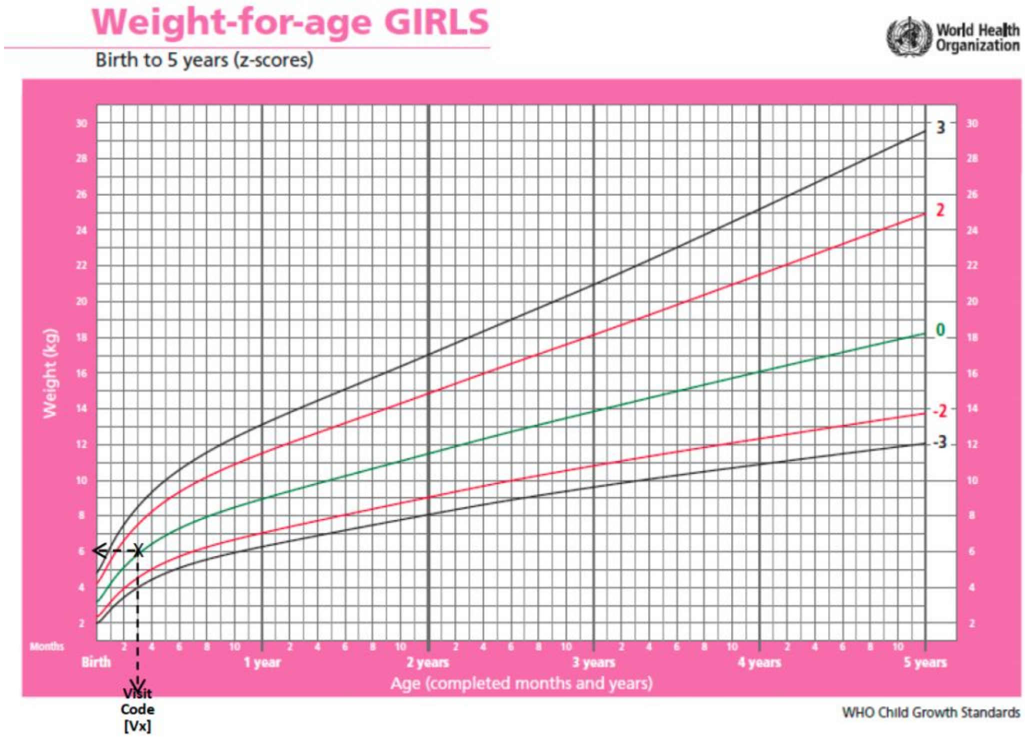

Reference Charts Adopted from the WHO Growth References: http://www.who.int/growthref/en/

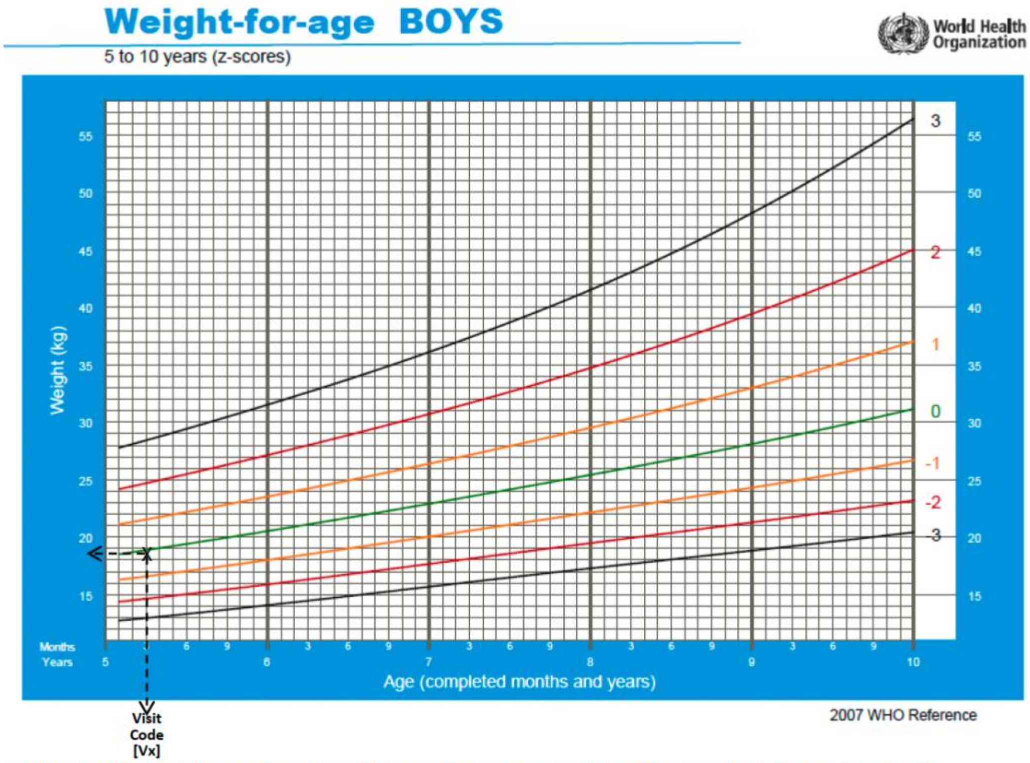

Reference Charts Adopted from the WHO Growth References: http://www.who.int/growthref/en/

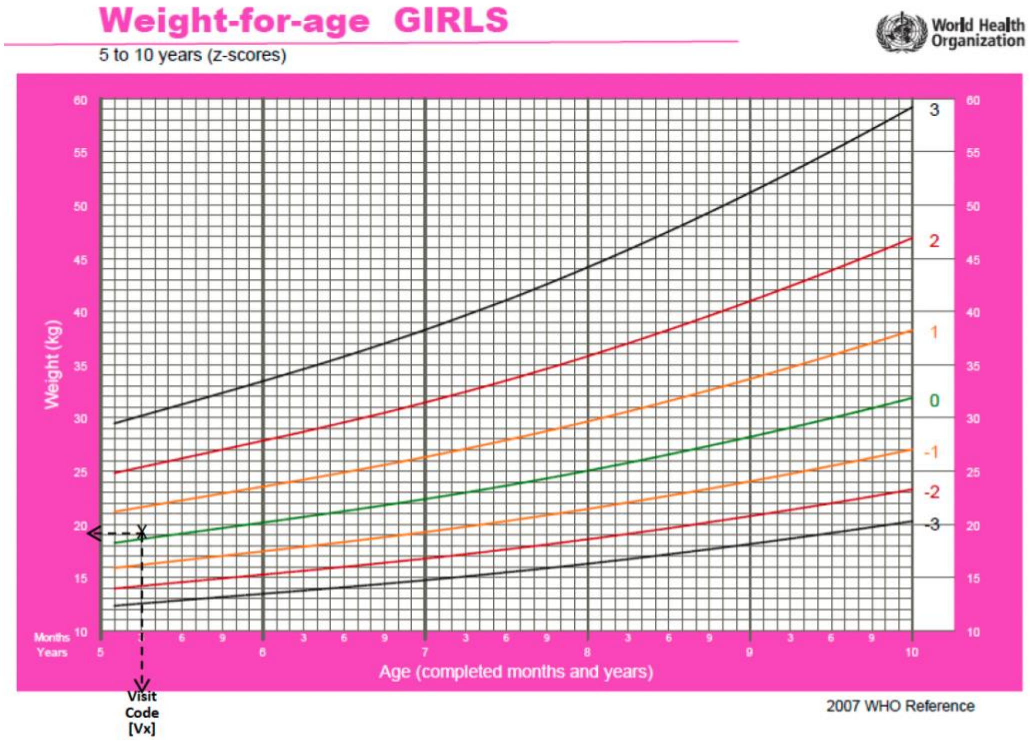

Supplemental Form 1	**National Malaria Control Program**<br>Bioko Island Malaria Elimination Program	

HOUSEHOLD QUESTIONNAIRE

**Instructions:** ☑=Tick Appropriate Response<br>**Format**: Time=(*hh:mm*) Hours; Date = (*dd-mmm-yyyy, eg. 11/JAN/2019)*	**Household Number:** ___.___.___.___.___.___.___.___.___.___.___
	**VISIT DATE:** ___.___/___.___.___/**20**___.___

Section A: Down-selection of Potential Participants at Household Level

☐Head of the Household OR ☐Designee	
**First name:**	
**Surname(s):**	
**Date of birth:**	___|___/___|___|___/___|___|___|___|
**Actual Age:**	Years; |___|___|/ Months; |___|___|
**Sex:**	☐¹Male ☐²Female
**Mobile #:**	|___|___|___|___|___|___|___|___|___|;☐¹Own ☐²Third Party

**Is there any household member meeting any of the following criteria in this household?**		**If Yes, please specify number of individuals, else enter N/A**				
**Age Group**		18mo - 9 Years	10-17 Years		18-50 Years	
**Inclusion (1)**:<br>Aged 18 months to 50 years at "estimated" time of consent/ assent	☐Yes<br>☐No					
**Sex**		M&F	M	F	M	F
**Inclusion (2)**: *Applied only for those in* ***(Inclusion 1)***<br>Male and Non-pregnant, non-Lactating female	☐Yes<br>☐No					
**Inclusion (3)**: *Applied only for those in* ***(Inclusion 2)***<br>Age-appropriate understanding and communication in Spanish	☐Yes<br>☐No					
**Inclusion (4):** *Applied only for those in* ***(Inclusion 3)***<br>Plan to stay in Bioko Island and willing to attend required appointments at CRC for at least two years	☐Yes<br>☐No					
**Inclusion (5):** *Applied only for those in* ***(Inclusion 4)***<br>Agree to be contacted by telephone or by home visit	☐Yes<br>☐No					
**Exclusion (1):** *Applied only for those in* ***(Inclusion 5)***<br>Not received investigational malaria vaccine in the last 5 years	☐Yes<br>☐No					

<table>
<tr><td># of Eligible Potential Household Members for Pilot Study are indicated in Row Exclusion (1)
Please, collect details shown in Section B for each potential participant Identified.
Interviewer Initials: |___|___|___| Date: I___I___I-I____I___I___I-I_____I_____I____I____I
Reviewer Initials: |___|___|___| Date: I___I___I-I____I___I___I-I_____I_____I____I____I</td></tr>
</table>

Section B: Demographic and Contact Details for individual Potential Participants

<table>
<tr><td colspan="3">PART 1: This section MUST be completed for ALL Potential Participants</td></tr>
<tr><td>Serial #:</td><td colspan="2">Potential Participant |___|___| of |___|___| In this household</td></tr>
<tr><td>Participant is:</td><td colspan="2">☐ [1]Adult ☐ [2]Child; Part 2 below MUST be completed with Parent/ Guardian details</td></tr>
<tr><td>First name:</td><td colspan="2"></td></tr>
<tr><td>Surname(s):</td><td colspan="2"></td></tr>
<tr><td>Date of birth:</td><td colspan="2">___|___/___|___|___/___|___|___|___|</td></tr>
<tr><td>Actual Age:</td><td colspan="2">Years; |___|___|/ Months; |___|___|</td></tr>
<tr><td>Sex:</td><td colspan="2">☐ [1]Male ☐ [2]Female</td></tr>
<tr><td>Mobile #:</td><td colspan="2">|___|___|___|___|___|___|___|___|___|;☐ [1]Own ☐ [2]Same as Parent / Guardian</td></tr>
<tr><td colspan="3">PART 2: This section must be completed if PARTICIPANT IS A CHILD (Else, tick here ☐ N/A)</td></tr>
<tr><td></td><td>Parent / Guardian (1): Same as</td><td>Parent / Guardian (2)</td></tr>
<tr><td>The adult is:</td><td>☐ [1]Parent ☐ [2]Guardian</td><td>☐ [1]Parent ☐ [2]Guardian</td></tr>
<tr><td>First name:</td><td></td><td></td></tr>
<tr><td>Surname(s):</td><td></td><td></td></tr>
<tr><td>Date of birth:</td><td>___|___/___|___|___/___|___|___|___|</td><td>___|___/___|___|___/___|___|___|___|</td></tr>
<tr><td>Actual Age:</td><td>Years; |___|___|/ Months; |___|___|</td><td>Years; |___|___|/ Months; |___|___|</td></tr>
<tr><td>Sex:</td><td>☐ [1]Male ☐ [2]Female</td><td>☐ [1]Male ☐ [2]Female</td></tr>
<tr><td>Mobile #:</td><td>|___|___|___|___|___|___|___|___|___|
☐ [1]Own; ☐ [2]Third party</td><td>|___|___|___|___|___|___|___|___|___|
☐ [1]Own; ☐ [2]Third party</td></tr>
</table>

Interviewer Initials: |___|___|___| Date of Interview: ___|___/___|___|___/___|___|___|___|

Reviewer Initials: |___|___|___| Date of Review : ___|___/___|___|___/___|___|___|___|

Section C: Disposition of Potential Participant in this household based on Exclusion Criteria (2):

<table>
<tr><td colspan="2">Two persons from the same household are already enrolled, or one person from the same age group</td></tr>
<tr><td>☐ Yes: Complete date of termination below</td><td>☐ No: Complete date of townhall visit below</td></tr>
<tr><td>Date of termination from the subsequent procedures</td><td>Scheduled Date of Potential Participant to Attend to the Townhall meeting</td></tr>
</table>

Date ___|___/___|___|___/___|___|___|___|	Date ___|___/___|___|___/___|___|___|___|

Investigator’s Initials: |___|___|___| Date of Decision: ___|___/___|___|___/___|___|___|___|
Coordinator’s Initials: |___|___|___| Date of Decision: ___|___/___|___|___/___|___|___|___|

References for Supplementary material

1. Malabo District Hospital Blood Bank: Data from healthy Equatoguineans
2. Bagamoyo Research and Training Centre, Ifakara Health Institute, Tanzania
3. TzSTG = Tanzania Standard Treatment Guidelines
4. US Food and Drug Administration. Guidance for industry: Toxicity grading scale for healthy adult and adolescent volunteers enrolled in preventive vaccine clinical trials. 2007. Available at http://www.fda.gov/BiologicsBloodVaccines/GuidanceComplianceRegulatoryInformation/Guidances/Vaccines/ucm074775.htm (last accessed 10 September 2010).
5. Saathoff E, Schneider P, Kleinfeldt V, GeisS, Haule D, et al. (2008) Laboratory reference values for healthy adults from southern Tanzania. Trop Med lnt Health 13: 612-625.
6. PfSPZ Vaccine IV trial in Mali 15 May 2014 Version 5.0
